# Occupational Stress and Catholic Priests: A Scoping Review of the Literature

**DOI:** 10.1007/s10943-021-01352-0

**Published:** 2021-08-15

**Authors:** Miguel Ruiz-Prada, Samuel Fernández-Salinero, Cristina García-Ael, Gabriela Topa

**Affiliations:** 1 Department of Social and Organizational Psychology, National University for Distance Education, Madrid, Spain; 2 Rey Juan Carlos University, Madrid, Spain

**Keywords:** Priests, Clergy, Occupational stress, Burnout

## Abstract

This study offers an exploratory review of the experience of stress and burnout syndrome among Catholic priests. Following Arksey and O’Malley's (Int J Soc Res Methodol 8(1):19–32, 2005, 10.1080/1364557032000119616)  protocol, a scoping study was conducted. Given the scarcity of studies found on the subject, a broad selection criterion  was used, which included quantitative, qualitative and mixed studies, literature reviews and comparative studies with other professions. The results reveal various risk factors: work overload among younger generations of priests, a sociocultural context that distrusts the clergy, neurotic, introverted, perfectionist and narcissistic personality styles, avoidant and complacent coping styles, living alone, not having sufficient support (especially from the Church authorities), excessive demands and lack of boundaries related to the priestly role and submissive obedience styles, among others. However, the studies reviewed also identified important protection factors: promoting optimism, an approach-based coping style and a collaborative way of resolving conflicts, frequent physical exercise, eating a balanced diet, finding time to rest, strengthening personal identity, social support (from parishioners, collaborators, colleagues, superiors) and leading an active spiritual life. Stress and burnout are associated with certain pathologies linked to smoking, alcoholism, obesity, diabetes, cardiovascular disease, anxiety and depression. Strengthening protective factors and minimizing the impact of risk factors would do much to improve the clergy’s occupational health.

## Introduction

Down the centuries, no era in humanity’s history has been free from stress, since each generation has had the challenge of facing a complex environment (wars, natural disasters, conflict, etc.). Stress has a positive dimension in that it has, throughout history, helped both individuals and societies to survive and make the most of their capacities. As such, stress helps increase resources and optimize productivity. Problems occur when this process of physical and psychological activation is sustained over long periods of time, since when this happens, resources are depleted, and performance suffers. It is therefore important to distinguish between “positive” stress or “eustress” and “negative” stress or “distress” (de Miguel et al., 2009; López, 2012).

One very common form of sustained stress or distress is occupational stress. According to person-environment fit theory, occupational stress occurs as a result of a misfit between the employee’s capacities and the demands of the environment in which they perform their job. This misfit may in turn be due to the fact that either the person’s aptitudes and capabilities do not correspond with those required by their job, or their job fails to satisfy their individual needs or expectations (López, 2012; Luceño et al., 2004).

Besides, recent research has demonstrated that clerical profession is related to complex work-related stress processes (Wells et al., 2012). When evaluating stress in clergy, it has been found that they experience stressors not only related to their careers, like counseling, teaching or guiding, but also effects of work stress expand to their personal lives. Even though recent research show that religious resources and emotional and spiritual well-being are related to positive coping strategies (Gall, 2000), there are scarce studies that evaluate stress processes among clergy.

Moreover, it should not be forgotten that the current labor market, which is increasingly complex and ever-changing, demands competitiveness, productivity and flexibility, which are experienced subjectively by employees as overload and pressure, giving rise to a wide range of occupational health-related pathologies, including absenteeism, burnout, occupational accidents and cardiovascular disease, among others (Gil-Lacruz & Izquierdo, 2004; Luceño et al., 2004; Serrano-Orellana & Portalanza, 2015). It has been demonstrated that clergy mental health may be improved via enhancing work resources. These measures have been related to positive outcomes such an improvement of spiritual well-being (Terry & Cunningham, 2020). One of the main reasons underlying current research is the fact that in many ways, clergy have been usually ignored notwithstanding they constitute a high-risk population (Terry & Cunningham, 2020). We believe that our research will help to identify the more common variables that impact in clergy occupational stress.

### Psychosocial Factors of Occupational Stress

With its demands for competitiveness, the labor market often causes individuals with a “type A” behavioral pattern to fall victim to distress. These individuals are characterized by excessive ambition, a strong need for achievement, being perfectionists, being impatient and competitive, feeling a sense of urgency, being too hard on themselves and on others, and experiencing a constant feeling of dissatisfaction. They also tend to overreact to stressful situations (García et al., 2015; Gil-Lacruz & Izquierdo, 2004).

Another internal aspect of the individual that has an impact on stress is the “locus of control”; in other words, the person’s belief regarding whether the situations in their life can be controlled by their own decisions (internal locus) or are controlled by external forces (external locus). Individuals who have (mainly) an external locus of control are more vulnerable to stress because they believe that their success and good performance depend on other people or situations (Asante & Affum-Osei, 2019).

In addition to these internal personality factors, there are also a number of other external stressors, which can be environmental, occupational and/or organizational in nature. These include: difficulties establishing a work-life balance, inflexible working hours, excessive tasks, repetitive tasks, difficulties linked to service beneficiaries, a feeling of having to be available round the clock, every day of the week, lack of limits in the performance of functions, excessive bureaucracy, structures which generate distrust, conflict with work colleagues, task ambiguity (unclear definitions), lack of positive challenging experiences, lack of recognition and supervision, poor occupational support and little participation in decision making (Durán, 2010; López, 2012).

One moderator variable of occupational stress is the attainment of rewards in the form of a good salary, social support, recognition and personal accomplishment, etc. Good rewards foster motivation, engagement (involvement in one’s work) and job satisfaction, minimizing the impact of work-related demands, whereas intense physical and/or psychological effort accompanied by few/poor rewards gives rise to occupational stress (Bakker & Demerouti, 2013).

### Burnout Syndrome and Boreout Syndrome

When stress is sustained over long periods of time, it may result in what is known as burnout syndrome. This syndrome manifests as intense physical and emotional tiredness, depersonalization, cynicism (distant and insensitive attitude toward service beneficiaries; the subject isolates themselves in order to protect themselves) and low self-esteem (the individual feels unable to do their job, has no sense of personal achievement and feels overwhelmed by having to deal with others). This syndrome is common among those who work with people and who feel that those to whom they provide a service are very demanding; these individuals often feel a vocation, have strong ethical convictions and work in a context with scarce financial and organizational resources (Maslach, 2017).

This syndrome was first described by Herbert Freudenberger in 1974. Since then, it has been thoroughly studied and specified. However, neither the ICD-10 nor the more recent DSM-5 consider it a specific disorder. Although it is a syndrome that affects many helping professions, including priests, from a medical-legal point of view, it still cannot be diagnosed as an occupational disease (Chirico, 2017b). However, the ICD-11, which will enter into force in January 2022, describes burnout as a syndrome related to chronic work stress, thereby providing legal coverage for the decisions made by occupational physicians (prescribing sick leave, for example) (WHO, 2018).

Health professionals must also distinguish between depression and burnout, two disorders which share certain symptoms. However, whereas burnout refers specifically to the work context, depression is a more general, context-independent disease. Moreover, cynicism toward aid recipients (patients, users, clients, etc.) is fundamental in burnout syndrome and is not present in depression (Chirico, 2017a).

Burnout syndrome correlates negatively with both engagement and employees’ perception of self-efficacy (Leiter & Maslach, 2017). Salanova et al. (2005) carried out a study in which they discovered two dynamics which, in a spiral form, hampered or fostered confidence in personal self-efficacy when attaining personal success: burnout (downward spiral model) and engagement (upward spiral model). University students who, in the past, had obtained good academic results positively assessed their capacities and were confident of future success. In other words, the greater the individual’s past academic success, the greater their perceived academic efficacy and the higher their levels of engagement. Burnout, on the other hand, was associated with a crisis of efficacy, further intensified by previous failures which anticipated future ones.

Some authors have recently started to talk also about boreout syndrome (Cabrera, 2014). This syndrome is characterized by three elements: the employee becomes bored because “they don't know what to do” during a large part of their working day or because the tasks they do are monotonous and tedious. The employee is disengaged: they do not identify with the company values and are not motivated, since they see no opportunity for advancement with the corresponding increase in responsibility, recognition and salary. Finally, the employee feels that not enough is demanded of them, since the organization assigns them few or irrelevant tasks, or those which do not challenge their personal skills and abilities. Employees themselves often perpetuate boreout syndrome by pretending to be overwhelmed by the amount of work assigned to them (“pseudo-burnout”), thereby avoiding any increase in workload: they fill their desk with documents, delay the completion of tasks and spend more time at work than their colleagues, etc. (Cabrera, 2014).

### Leadership Styles and Occupational Stress

The type of leadership exercised by management in an organization or institution is another key variable to be taken into consideration. Traditional approaches to leadership support an “individualistic” understanding of this concept. Nevertheless, Social Identity Theory (Tajfel & Turner, 1979) posits that leadership can be better understood as “something to do with the us” than as “something to do with the self.” From this perspective, Haslam et al. (2011) argue that good leadership is that which is able to build an identity shared by both leader and followers. Leadership could therefore be defined as the “process of influencing other people in ways that motivate them to contribute to the achievement of collective goals” (p. 79). One key term in this approach is “influence,” since what leaders seek is to make followers “want to do” what is asked of them.

Thus, organizations which engage in a traditional, pyramid-type leadership, in which management rigidly dictates how things should be done and employees follow instructions with no channel for making suggestions, do not seem the best positioned to foster engagement and job satisfaction. More inclusive models of leadership, however, based on the leader’s ability to influence and include, and which seek to promote a flatter organizational structure, facilitating teamwork and networking, seem more conducive to proactive intrapreneurial behavior, which generates satisfaction and well-being among those who engage in it (Moriano et al., 2014). Let us remember that occupational stress occurs when the job fails to satisfy the employee’s needs or expectations, and when the employee does not feel supported by their leader (Serrano-Orellana & Portalanza, 2015).

In this sense, analyzed the modulating role of job engagement in the relationship between stress and job satisfaction. The sample comprised 779 professional soldiers from the Spanish Army. In general, the results indicated that, when levels of the two dimensions of job engagement analyzed (psychological identification and feelings of duty-obligation toward the job) were high, stress did not appear to affect job satisfaction. However, occupational stress did have an impact when job engagement was low.

### Occupational Stress and Burnout Among Priests

Being a priest in today’s world involves new challenges which can be highly stressful: globalization (fast-paced social, economic and cultural changes); secularization (loss of influence by religion and its institutions); large-scale decrease in religious practice; the importance of personal fulfillment (freer and more individualistic spiritual searches); priests’ loss of status and standing (contradiction between the theological and sociological images of the clergy: whereas before they were powerful and respected, they are now considered marginalized, out-of-date and obsolete); drop in the number of priests and the general aging of the clergy; the promotion of women; and increasingly urban, technological and democratic societies, etc. (Cozzens, 2003; López, 2012; Lowney, 2018).

Burnout is a concern among the clergy. This was the conclusion drawn by López (2009) in her doctoral thesis. In a sample of 881 Catholic priests in Latin America, this author found that three out of every five suffered from this syndrome, in either its intermediate or advanced phase. Moreover, one out of every four suffered from severe burnout. A priest suffering from this disorder was found to be seriously damaged or simply unable to help others adequately.

Consistently with the approaches described above, and under the title “Los curas están estresados” (Priests are stressed), Vidal (2012) wrote an article in the newspaper “El Mundo” outlining the conclusions reached by participants at a conference held at the Salesian Pontifical University in Rome in March 2012 (“Preti sul lettino. Agio e disagio del servizio pastorale del clero”; “Priests on the Couch. Well-being and distress in pastoral work”). Some causes of stress experienced by priests (which on occasions becomes chronic and turns into burnout syndrome) include: their enormous workload (which increases as the number of clergy diminishes and they all grow older); being at their congregation’s disposal at any time of the day or night; feeling like mere “dispensers of sacraments”; conflicts with fellow clergy (young-old; progressive-conservative); the excessive demands of some parishioners; increases in the number of those who are indifferent to priests, distant from them and even overly critical toward them; and living alone (not receiving enough affection) and working alone. The experts who met at the aforementioned conference identified three elements for coping with stress: prayer, priestly brotherhood (as a source of aid, refuge and consolation) and, when necessary, asking for help from a mental health specialist.

Two speakers at the conference held in Rome, Crea and Mastrofini, had previously (2010) published the work “Preti sul lettino” (“Priests on the Couch”). This book presented the results of recent research into the Italian clergy, which coincide with those presented so far in this paper: the clergy as a group is aging, priests live and work alone, the workload is increasing, and they feel dissatisfied and tired. Alongside these statistical data, the book also presented qualitative information provided by psychotherapists working with priests, who identified a wide variety of different problems: they feel indispensable, they do not feel understood, they cannot stand their collaborators, and they feel that, while their job is to provide aid, they have little training in emotional intelligence and social skills, etc.

In relation to burnout and boreout syndromes, seen as two opposing manifestations of occupational stress, López (2012) proposes distinguishing between three different types of priest when dealing with the challenges posed: those who are always exhausted, which results in a chaotic lifestyle (in terms of rest, diet, exercise, prayer and study, etc.) and a feeling of failure in their lives (burnout syndrome); those who minister to their flock without much enthusiasm, never fully developing as priests and denying their congregation the benefit of their talents and energy (boreout syndrome); and those who are able to do their job fully without detriment to their health, honoring their commitment faithfully with complete serenity.

One variable which has a notable influence on stress among priests is their experience of the “priestly role.” Gnani (2013) claims that role conflicts are very common in priestly life and can cause high stress levels. A role conflict implies (at least) the presence of three elements: the individual (the priest himself), the group (the congregation to which he is assigned) and the organization (the diocese). To assume that role-related problems can be attributed solely to dynamics occurring within the individual (“Father X has a nervous temperament and finds it hard to accept change”), the limits of the institution (“the bishop is far away and doesn't understand what he is asking”) or the characteristics of the group (“it's a difficult parish; it's a very cold congregation”) is to see only part of the picture.

### Study Aims

According to data published by the Spanish Episcopal Conference (2017), the Catholic Church in Spain currently has 16.334 diocesan priests and 6.745 priests belonging to religious orders who engage in pastoral work (23.079 priests in total). For its part, the Press Office of the Holy See (2019) set the number of Catholic priests in the world today at 414.582 (data from 2017). However, despite the large number of clergymen within the Catholic Church, hardly any studies have sought to explore how this group functions in terms of Social Psychology and Organizations, particularly in relation to experiences of stress.

It is true that, over recent years, the ecclesiastical authorities have expressed a growing interest in the contributions made by psychology to our understanding of human maturity and have begun to use this knowledge in both the initial training provided to seminarians (Congregation for the Clergy, 2016) and the ongoing training provided to priests (John Paul II, 1992). Nevertheless, a detailed search of the literature revealed only one rigorous study focusing on a sample of Spanish priests. This study was published by Gómez (2009) 10 years ago and emerged from the doctoral thesis carried out by the author, which explored psychological health and human fulfillment in a group of 770 priests and 753 lay people (comparison group) from all the autonomous communities in Spain.

The present study, therefore, aims to make a modest contribution to this neglected field of study, namely that of Catholic priests, from the perspective of Psychology. Due to the novelty and the lack of research in this field, following Arksey and O’Malley’s (2005) suggestions, we conducted a scoping review. Scoping study tries to address topics and review literature but not to assess the quality of included studied (Arksey & O’Malley, 2005). Besides, it has been stated that scoping studies are a rigorous and transparent method for mapping areas of research. Moreover, scoping review may be used for illustrating the volume, nature and other circumstances around the field of research. Last, this method may be seen as a method in its own right for contributing to the publication and dissemination of research findings. Having said this, the following aims are proposed:

1. To conduct an exploratory review of papers published in scientific journals which include any of the variables which influence the experience of work-related stress among Catholic priests.
2. To systematize and categorize the main results of the variables over work-related stress among Catholic priests.
3. To construct a basic theoretical framework for future research in this field in Spain.

## Method

As mentioned above, scoping review method purposes a process which is documented in detail to enable the study to be replicated (Arksey & O’Malley, 2005). For this purpose, we followed the steps of (Arksey & O’Malley, 2005). The first stage of the process is to identity the research question. In our case, the research question was which are the factors related to stress among clergy? Scoping review literature suggests that researchers may not place strict limitations on search terms or relevant studies. In Table 1, steps of scoping review process may be seen.

**Table 1:** Stages of the scoping review process

Stage	Important issues
1. Identifying the research question	Define parameters and considering the implications
2. Identifying relevant studies	First aspect is to be as comprehensive and wide as possible. Define appropriate terms, key concepts and define the search strategy
3. Study selection	It is recommended to pick a large number of studies for, subsequently, eliminate those that don’t address the central issue of our research
4. Charting the data	Perform a narrative review. Decide which information and variables are important to be considered from the primary studies
5. Collating, summarizing and reporting the results	Include only a small percentage of the results reviewed. Scopus review aims to present an overview of all the material. Scoping review does not seek to assess quality or evidence of the material

Extracted from Arksey and O’Malley (2005)

After defining the research question, we moved into the second stage of scoping review proposal, which is to identify relevant studies. This stage comprises the selection of databases, the selection of right terms and to pilot the search strategy. Besides, it comprises the strategy for limiting the abundance of results. When introducing the concept of “burnout,” the PyscInfo Thesaurus (American Psychiatric Association—APA) requests that the term “Occupational Stress” be used more precisely. After several “trials” carried out in that same database, the following terms were chosen for use in all databases: (“Occupational Stress” OR “Stress”) AND (“Clergy” OR “Priests”). In each database, a sufficiently “broad question” was posed in order to ensure a significant number of records were identified. In other fields in which there is an abundance of empirical evidence, it would have been possible to have been more “restrictive,” limiting the target variables to a much greater extent (Urrútia, 2006), besides this is coherent with scoping review method.

Indeed, the ambiguity of the concepts “Clergy” and “Priests” (which include ministers from both other Christian denominations and other religions) was deliberate, since preceding them with the term “Catholic” resulted in almost no records being identified.

The search identified the following records: 181 studies in PsycInfo, 477 in Scopus, 258 in Web of Science, 116 in PubPsych and 173 in PubMed, making a total of 1,205 references.

Due to the abundance of literature on this topic, we decided to enter the following “limiters” into the aforementioned databases: only papers published in scientific journals, only papers published from the year 2000 onwards, and only papers written in English or Spanish. The year 2000 was established as a cutoff date because it was from that time onwards that the majority of research was conducted, and because all studies from that time onwards shared the same sociocultural context (i.e., the same environmental stressors). This is the third stage of Arksey and Malley’s (2005) suggestions for scoping review method.

Again, a “broad” criterion was used here in order to ensure a sufficient number of records were identified. The results were as follows: 80 studies in PsycInfo, 83 in Scopus, 48 in Web of Science, 85 in PubPsych and 99 in PubMed, reducing the total number of records to 395.

After reading the “Title” and the “Abstract” (and in some cases the “Method” section, particularly as regards the information given about participants), we selected those scientific papers which focused mainly on stress and referred to Catholic priests, either specifically, as part of a study on ministers from other faiths or in comparison with other professions.

**Fig. 1:**
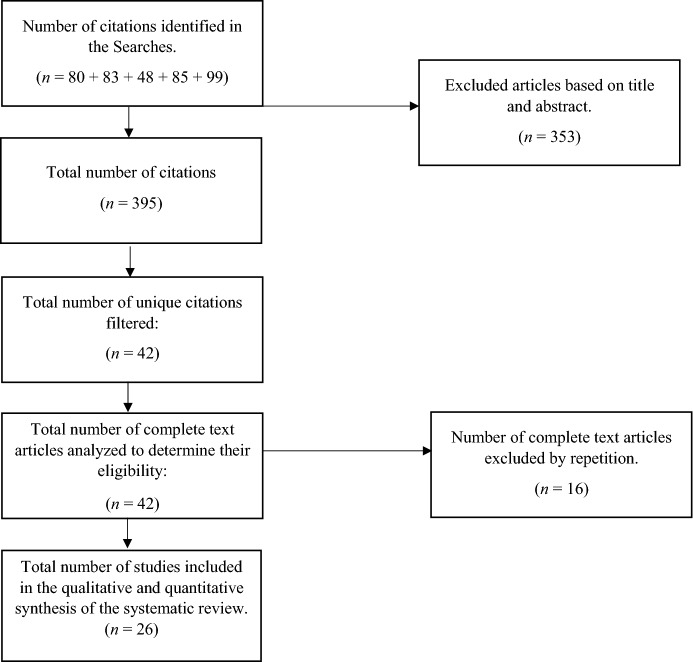
Flow diagram of the information through the different phases. Adapted from Urrutia and Bonfill (2010)

Although the search was laborious, it was considered the best way of “recovering” all studies exploring stress among Catholic clergy. The final sample of studies comprised 14 scientific journal papers in PsycInfo, 10 in Scopus, 8 in Web of Science, 3 in PubPsych and 7 in PubMed, giving a total of 42. Once all duplicates had been removed, the final list comprised 26 scientific papers. The article selection criteria may be seen in Fig. 1.

Moreover, the papers selected referred to both priests belonging to religious orders and diocesan priests. In some cases, comparisons were made between the two situations, while in others no distinction was made at all, even though the variables “loneliness” and “relationship with authorities” are experienced very differently by the two types of clergies.

## Results

Following (Arksey & O’Malley, 2005) scoping review steps, we moved into step four. This step is related to charting data. Authors propose an approach akin to a narrative review (Pawson, 2002). This is a very important step of our revision because it comprises the decision of selecting the important information that should be recorded for the selected studies. To this end, and following a narrative approach, there are six fields that should be addressed: (a) author(s) and year of publication, (b) study populations, (c) aims of the study, (d) methodological aspects, (e) outcomes measures and (f) important results (which may be seen as step five).

Conducting our review, the first difficulty identified in the selected studies was the need to adapt the measurement instruments to the language and vocational profile of Catholic priests. For example, the Maslach Burnout Inventory (Maslach et al., 1996) was reformulated in different studies, with the language being modified and items added to better reflect priests’ experiences (e.g., the word “client” was removed from the questionnaires when referring to parishioners).

Some studies went even further and substituted the three components of burnout syndrome proposed by Maslach (emotional exhaustion, depersonalization and loss of personal accomplishment) for a two-component model measuring the balance between negative and positive affect. This is the proposal made by the Francis Burnout Inventory. Thus, when priests have high levels of negative affect and low levels of positive affect, this is linked to emotional exhaustion and poor work-related psychological health. On the other hand, if they have high levels of positive affect and low levels of negative affect, the results are associated with feelings linked to satisfaction with their ministry (Francis et al., 2017).

After reading the selected texts, and aiming to systematize our findings, the results obtained were grouped into the following variables, all of which affect occupational stress and burnout among Catholic priests. For systematizing the exposition, we have organized the results in the following categories: (a) sociodemographic variables, (b) psychological factors and coping styles, (c) living conditions, (d) sociocultural context, (e) demands of the job, (f) organizational variables, (g) comparative studies and (h) stress, burnout and health. For a complete revision of the results, Table 2 may be seen.

**Table 2:** Articles included in the qualitative review

No.	Authors and year	Size of sample	Sampling process	Assessment instrument and variables included	Design used	Results
1	Adams et al. (2017)	Helping professions: social workers, teachers, Counselors, police, emergency personnel, clergy	Computerized search using NCBI PubMed, PsycINFO, ProQuest Dissertations, and the Cochrane Library for original research published in the English language that concerned burnout in helping professions	Maslach Burnout Inventory (MBI): comparison of burnout in clergy with other helping professions	Literature Review	**Emotional exhaustion** (EE): = social workers, counselors and emergency personnel < teachers and police **Depersonalization** (DP): > social workers and counselors = teachers < emergency personnel and police **Personal Accomplishment** (PA): > counselors = social workers and teachers < police and emergency personnel
2	Beebe (2007)	343 ministers from different confessions (9% Catholic). New York 223 men, 66 women, 1 unspecified Mean age: 54 years old	Clergy (*N* = 1.100) were contacted by mail with a packet including letters, an approved protocol, a demographic sheet, the five randomly ordered questionnaires and a stamped, self-addressed return envelope. The return rate was 31.2%	Differentiation of Self and Role–Clergy Version (DSR-C) (Beebe, 2004) Maslach Burnout Inventory–Educators Survey (MBI-ES) (Maslach et al., 1996) Occupational Stress Inventory–Revised (Osipow, 1998) Thomas–Kilmann Conflict Mode Instrument (TKI) (Kilmann & Thomas, 1977)	Correlational	**Significant multivariate relationships**: MBI-ES and role overload: *F*(3, 286) = 46.45, *p* < .001 MBI-ES role ambiguity: *F*(3, 285) = 31.75, *p* < .0001 MBI-ES and DSR-C: *F*(3, 284) = 14.72, *p* < .0001 MBI-Es and TKI: *F*(25, 1041.6558) = 4.72, *p* < .0001
3	Büssing et al. (2013)	425 Catholic priests. Germany Mean age not provided. Between 40 and 60 years old	The priests were informed about the study by the personnel manager of the dioceses and invited by a separate letter from the authors to participate in the study. 425 of the 998 people contacted completed the questionnaire (participation rate of 43%). Among the respondents, 297 answered the print version (70%), and 128 preferred the online form (30%)	Spiritual Dryness Scale (Büssing et al., 2013) MBI (Maslach & Jackson, 1996) Brief Symptom Inventory (BSI-18) (Derogatis, 2000) Perceived Stress Scale (PSS) (Cohen et al., 1983) Revised Life Orientation Test (LOT-R) (Scheier et al., 1994) The Sense of Coherence Scale (SOC) (Antonovsky, 1987) Self-efficacy Scale (GSE) (Hinz et al., 2006) Utrecht Work Engagement Scale (UWES) (Schaufeli et al., 2002) Diener’s Satisfaction with Life Scale (SWLS) (Glaesmer et al., 2008) Daily Spiritual Experience Scale (DSES) (Underwood & Teresi, 2002) SpREUK-P Questionnaire: specific spiritual practices (Büssing et al., 2005)	Correlational	**Significant correlations with spiritual dryness** (*p* < .01) Daily spiritual experiences: *r* = − .*66* Active religious practices: *r* = − .45 Gratitude: *r* = − .39 EE-MBI: *r* = .46 DP-MBI: *r* = .45 PA-MBI: *r* = .44 Anxiety: *r* = .38 Self-efficacy: *r* = − .30 Optimism: *r* = − .41 Pessimism: *r* = .33 Satisfaction with life: *r* = − .43 Sense of coherence: *r* = − .48 Work engagement: *r* = − .43 Perceived stress: *r* = .466
4	Büssing et al. (2017)	3.824 Catholic priests. Germany Mean age not provided. Between 45 and 55 years old	Participants were recruited from among the Catholic priests of 22 of the 27 German Dioceses. All were informed about the study by the personnel managers of the dioceses and invited by a separate letter from the study authors to participate in the study. Participants chose between a pencil and paper version and an online questionnaire	Spiritual Dryness Scale (Büssing et al., 2013) MBI (Maslach & Jackson, 1996) Brief Symptom Inventory (BSI-18) (Derogatis, 2000) Perceived Stress Scale (PSS) (Cohen et al., 1983) The Sense of Coherence Scale (SOC) (Antonovsky, 1987) Self-efficacy Scale (GSE) (Hinz et al., 2006) Daily Spiritual Experience Scale (DSES) (Underwood & Teresi, 2002) Big Five personality factors (Gerlitz & Schupp, 2005) Social Support Questionnaire (Fydrich et al., 1999) Loneliness (Russel et al., 1984)	Correlational	**Significant correlations with spiritual dryness** (*p* < .01) Daily spiritual experiences: *r* = − .52 Depression: *r* = .49 Anxiety: *r* = .36 Perceived stress: *r* = .44 Burnout: *r* = .49 Loneliness: *r* = .33 Neuroticism: *r* = .31 Sense of coherence: *r* = − .33 Self-efficacy expectation: *r* = − .49
5	Ferguson et al. (2015)	539 ministers from different confessions (5% Catholic) USA Mean age 53 years old	Authors use data from the 2008/9 US Congregational Life Survey; a national sample of clergy from multiple religious traditions	Body Mass Index (BMI) Clergy Occupational Distress Index (CODI) (Frenk et al., 2013) Sociodemographic variables and religious tradition	Correlational	32% of ministers are obese **Binary logistic regression**—obesity probability (*p* < .05): Hours worked per week (1.02), occupational distress (1.14), two jobs (2.91), protestants with family (1.83), sabbatical (0.48) support group (0.57)
6	Francis and Crea (2015)	155 Catholic priests Italy Mean age 46 years old	Catholic priests who participated in programs on psychology and spirituality were voluntarily invited to answer the questionnaire: 63% Italians—37% other countries; 56% diocesan priests—44% religious priests	Francis Psychological Type Scales (FPTS) (Francis, 2005 Francis Burnout Inventory (FBI) (Francis, 2005)	Correlational	**Satisfaction in ministry**: Ministry gives real meaning and purpose to their life (83%) Achievements in their ministry (77%) Their pastoral ministry has a positive influence on people’s lives (77%) and faith (76%) Their ministry is really appreciated by people (74%) **Emotional exhaustion in ministry:** Fatigue and irritation are part of their daily experience (28%) Lack of personal support for them in their ministry (23%) Their humor has a cynical and biting tone (20%)
7	Francis and Crea (2018)	95 Catholic priests and 61 sisters Italy Mean age 50 years old	Catholic priests and sisters who participated in programs on psychology and spirituality were voluntarily invited to answer the questionnaire: 33 non-graduated—123 graduated	Francis Burnout Inventory (FBI) (Francis, 2005) Francis Psychological Type and Emotional Temperament Scales (FPTETS), a development of the Francis Psychological Type Scales (FPTS) (Francis, 2005) Oxford Happiness Questionnaire (Hills and Argyle 2002)	Correlational	**Positive items:** Agree that life is good (93%) Always committed and involved (92%) Experiencing joy and elation (90%) Intensely interested in people (90%) Life is very rewarding (86%) **Negative items:** Difficulty to make decisions (47%) Imbalance between desire and reality (31%) Absence of happy memories of the past (27%) Lack of control in their life (26%) **Significant correlations** (*p* < .001): Personal happiness with extraversion (*r* = .31), emotionality (*r* = − .45), emotional exhaustion (*r* = − .59) and satisfaction in ministry (*r* = .61) Satisfaction in ministry with emotionality (*r* = − .39) and emotional exhaustion (*r* = − .57)
8	Francis et al. (2017)	155 Catholic priests Italy Mean age 46 years old	Catholic priests who participated in programs on psychology and spirituality were voluntarily invited to answer the questionnaire: 63% Italians—37% other countries; 56% diocesan priests—44% religious priests	Francis Burnout Inventory (FBI) (Francis, 2005) Purpose in Life Scale (PILS) (Robbins and Francis, 2000)	Correlational	More **time spent in prayer** was associated with lower scores of emotional exhaustion: 10–30 min = 28.7; more than 1 h = 23.2 **Correlations** between purpose in life and satisfaction in ministry (*r* = .58) and emotional exhaustion (*r* = − .44) **Multiple regression** of PILS on emotional exhaustion (*β* = − .84; *t* = − 8.4; *p* < .001) and interaction with satisfaction in ministry (*β* = .01; *t* = 5.8; *p* < .001): mitigation effect of satisfaction in ministry
9	Francis et al. (2004)	1.482 Catholic priests England and Wales Mean age not provided 98% of the sample above 30 years old	A total of 3581 questionnaires were mailed to all regular and secular priests in England and Wales involved in parochial ministry (response rate of 41%)	Modified form of the Maslach Burnout Inventory (Rutledge & Francis, 2004) Eysenck Personality Questionnaire (Eysenck and Eysenck, )^1975^	Correlational	**Significant correlations (***p* < .001) between EE and DP (*r* = 0.62); EE and PA (*r* = − 0.4223); DP and PA (*r* = − 0.52) **Significant correlations (***p* < .001) between age and EE (*r* = − .16) and DP (*r* = − .14) **Significant correlations** (*p* < .001) between EE and Neuroticism (*r* = .51), DP and Neuroticism (*r* = .45), PA and Extraversion (*r* = .38) **Comparing** with Anglican parochial clergy, Roman Catholic parochial clergy presents higher scores of EE (24.0–22,3) and DP (23.2–19.9), and lower scores of PA (23.4–24.7)
10	Francis et al. (2007)	1.482 Catholic priests England and Wales Mean age not provided 98% of the sample above 30 years old	A total of 3581 questionnaires were mailed to all regular and secular priests in England and Wales involved in parochial ministry (response rate of 41%)	Modified form of the Maslach Burnout Inventory (Rutledge & Francis, 2004) Eysenck Personality Questionnaire (Eysenck and Eysenck, 1975) Companion animals: «Which of the following pets do you own?» Options: bird, cat, dog, fish, other	Correlational	Not significant correlations between owning a cat and burnout Positive correlation between owning a dog and EE (*r* = .060, *p* < .05) and owning a dog and DP (*r* = .070, *p* < .01)
11	Frick et al. (2015)	8.574 Catholic pastoral professionals (48% priests) Germany 75% men, 25% women Mean age not provided	Participants were recruited from Catholic Priests and pastoral ministry workers from 22 of the 27 German dioceses. The personnel manager of the dioceses informed them of the study with a letter from the authors inviting them to participate. Answers, anonymous, were given through either a pencil and or an online version	Cohen’s Perceived Stress Scale (PSS-10) (Cohen et al., 1983) Brief Symptom Inventory (BSI-18) (Franke, 1997, 2000; Franke et al., 2011) Spiritual Dryness Scale (SDS) (Büssing et al., 2013) Daily Spiritual Experiences Scale (DSES) (Underwood and Teresi, 2002; Underwood, 2011) General Self-Efficacy Scale (SES) (Schwarzer and Jerusalem, 1995) Diener’s Satisfaction with Life Scale (SWLS) (Diener et al., 1985)	Correlational	**Female–male comparison:** + Women had lower depression scores than men (2.43–2.63), higher self-efficacy expectation (28.66–28.28) and higher life satisfaction (5.45–53.9) - But higher perceived stress (15.79–15.26) and anxiety (2.90–2.72) **Age comparison**: + Older persons had the lowest scores for anxiety (2.26), depression (2.19), stress perception (14.23) and the highest life satisfaction scores (5.60) - But also, the lowest self-efficacy scores (27.66) and the highest somatization (2.58) **Profession comparison**: Priests had the worst scores in depression (2.88), somatization (2.42), stress perception (15.80), self-efficacy expectation (27.65), life satisfaction (5.30) and spiritual dryness (2.21) **Significant correlations** (*p* < .001): Anxiety with depression (*r* = .645), with somatization (*r* = .608), with stress perception (*r* = 552) Depression with somatization (*r* = .511), with stress perception (*r* = .511), with life satisfaction (*r* = − .50) Daily spiritual experience with spiritual dryness (*r* = − .55)
12	Greene et al. (2017)	103 Catholic diocesan priests USA Mean age 58 years old	3500 priests living in the USA were randomly selected from all listings in The Official Catholic Directory and e-mailed a request to participate in an online study of Roman Catholic clergy on psychological distress that included sexual identity as one study variable. Of those contacted, 135 responded, with 103 surveys completed (3% of the overall sample)	K6 (Kessler et al., 2003): measure non-specific psychological distress (6 items) Perceived Stress Scale (PSS) (Cohen et al., 1983) Perceived Social Support Scale (Walen and Lachman, 2000) Fear of Compassion Scale—From Others Subscale (FCS-O) (Gilbert et al., 2011) Three open-ended essay questions that appeared at the end of the questionnaire	Mixed	**Significant correlations** (*p* < .01): Psychological distress with social support (*r* = − .37), with Fear of compassion from others (FC-O) (*r* = .40) Social support with FC-O (*r* = − .28), with Stress (*r* = − .45) **Multiple linear regression**: psychological distress was significantly predicted by stress (*B* = .64, *t* = 8.40, *p* < .001), FC-O (*B* = .25, *t* = 3.36, *p* < .001) and gay compared to heterosexual-identity, (*B* = .19, *t* = 2.70, *p* < .01) **ANOVA**: significant difference between heterosexual and gay psychological distress (*M* = − 1.38, *p* < .05)
13	Isacco et al. (2014)	15 Catholic priests USA Between 29 and 76 years old	This study utilized snowball sampling techniques. From the priest who completed the pilot interview, each participant was asked if they knew other priests that may be willing to participate in the study until 15 participants were achieved	Consensual Qualitative Research (CQR) methodology: attitudes and behaviors related to mental health help-seeking, stress, burnout and self-care strengths and supports of priests religious/spiritual aspects of priest health mental health professionals’ competencies for working with priests	Qualitative	**Attitudes and behaviors about mental health help-seeking**: Counseling helps, heals and supports priests; helps to deal with specific problems (e.g., alcoholism, depression, anxiety and stress); enhances strengths and self-knowledge; was recommended by friend, spiritual director, or another priest **Barriers to priests seeking mental health services:** No perceived need for mental health services (enough supports; own pride). Stigma of help-seeking. Concerns about how diocese would perceive the priest seeking mental health services **Advice from priests to mental health professionals**: Priests have unique stressors (social, work, and spiritual stressors). High expectations stressors. The importance of prayer for a priest. Interventions need to be congruent with priest’s personality
14	Isacco et al. (2016)	15 Catholic priests. USA Mean age 47 years old	This study utilized snowball sampling techniques. Starting with the priest who completed the pilot interview, each participant was asked if they knew other priests that may be willing to participate in the study until 15 participants had been obtained	Consensual Qualitative Research (CQR) methodology. This article focuses on “religious/spiritual aspects of priests’ health”	Qualitative	**Dynamic relationship with God** increases positive and decreases negative emotions; transforms; empowers; enables authenticity; creates balance; decreases stress; strengthens relationships and connection **Promise of obedience**: (-) internal conflict and disrupts relationships; ( +) increases positive emotions, decreases stress and strengthens relationships and connection **Promise of celibacy**: (-) loneliness, depression and biological desires; ( +) increases focus on vocation
15	Joseph et al. (2011)	511 Catholic priests India Mean age 43 years old	Priest delegates of the dioceses of South India, distributed and collected the questionnaires. Most of them personally visited the priests, explained the nature of the research, and distributed the questionnaires. 800 questionnaires were distributed (540 answered = 67.5%; 511 completed = 63.9%)	Maslach Burnout Inventory (MBI) (Maslach, 1982) Utrecht Work Engagement Scale (UWES) (Schaufeli et al., 2002) NEO Five-Factor Inventory (NEO-FFI) (Costa & McCrae, 1992)	Correlational	**Significant correlations** (*p* < .01): Conscientiousness with EE (*r* = − .33), with DP (*r* = − .31), with PA (*r* = .35), with Engagement (EN) (*r* = .44) Agreeableness with EE (*r* = − .51), with DP (*r* = − .56), with PA (*r* = .32), with EN (*r* = .48) Extraversion with EE (*r* = − .34), with DP (*r* = − .32), with PA (*r* = .33), with EN (*r* = .48) Neuroticism with EE (*r* = .53), with DP (*r* = .50), with PA (*r* = − .44), with EN (*r* = − .50)
16	Kane (2017)	18 Catholic priests USA No mean age was reported All priests above 45 years old	This study utilized snowball sampling techniques. Three priests known to the primary investigator were contacted. They were asked to participate in a semi-structured interview and if they would be willing to recommend the names of other priests who would want to participate in a qualitative interview: 21 priests were contacted; 18 participated (expected time commitment: 30–90 min)	Semi-structured interview: (1) describe the physical changes you have noticed as you age. (2) Have you noticed any cognitive changes as you age? (3) Have any physical changes required you to alter your ministerial activities? (4) In what ways are you taking prudent care of yourself? (5) Can you describe at least five ways in which you take care of your health? (6) What causes you stress in your priesthood? (7) What do you do for relaxation? (8) Describe your prayer life outside of liturgy and public services. (9) Which best describes you: (a) Every day is a new and wonderful adventure; (b) I am satisfied with my life, but sometimes the days are too long; or (c) I look forward to retirement	Qualitative	**Physical and cognitive changes of aging**: difficult to maintain the necessary amount of energy required for their ministerial demands; desire to continue learning vs. memory impairment **Stress in my life**: parochial demands, loneliness, lack of support **Self-care resources**: exercise and diet, routine medical care, relaxation and private prayer activity
17	López et al. (2014)	881 priests Mexico, Central America and the Caribbean Mean age 46 years old	Convenience sample Instrument was sent to each diocese, together with a letter of presentation. Response rate of 96.60%	The *Maslach Burnout Inventory* (MBI-22; Maslach & Jackson, 1981) The *General Health Questionnaire* (GHQ-28) = somatic symptoms, anxiety and insomnia, social dysfunction and severe depression The CAGE test (Ewing, 1984) (alcohol consumption) Tobacco use was assessed by requesting participants to indicate how many cigarettes they smoked per day	Correlational	There are no statistically significant differences on the prevalence of the syndrome by countries **Likelihood of a priest being free of** • Burnout 39.62% • Showing a moderate level of this disorder 60.38% • The exhaustion and the depersonalization dimensions appear to be the most determinant in the development of the syndrome **Burnout and health level** (Canonical correspondence) = •Strong association between exhaustion and depersonalization belonging to the MBI •Relation between anxiety and insomnia of the ghq-28 and the depersonalization and exhaustion dimensions, •Negative correlation between personal accomplishment and depression **Lifestyle** Exhaustion and smoking .36 *p* = .007* Depersonalization and the use of both alcohol .37 *p* = .001 and tobacco .33, *p* < .001*
18	Man-Ging et al. (2018)	499 German Roman Catholic priests Germany No mean age reported All priests above 65 years old	Priests were informed through an invitation letter about the purpose of the research. Cross-sectional study	Brief Symptom Inventory (BSI) (Derogatis,^2001^ ) = Somatization (6 items), Depression (6 items) and Anxiety (6 items) Coping Inventory Stressful Situations (CISS) (Endler and Parker 1990). = Task-Oriented Coping, Emotion-Oriented Coping, Avoidance-Oriented Coping Religious Coping Scale (RCOPE) (Pargament et al. 2011) = Positive and Negative Identification with Priestly Role (RI) = from 1 up to 5 to the question How strongly do you identify with your life as a priest?	Correlational	Predictors of Psychosomatic Symptoms = 34% of **depressive symptoms** variance was explained by • Low Identification (*β* = − .23, • Low Task-Oriented Coping (*β* = − .164, • High Emotion-Oriented Coping (*β* = .47). All *p*_s_ > .001 29% of **anxiety symptoms** variance was explained by: • Emotion-Oriented Coping (*β* = .49) • Low Identification (*β* = − .12) • Low Task-Oriented Coping (*β* = − .102). All *p*_s_ > .001 13% of **somatization** variance was explained by = • Emotion-Oriented Coping (*β* = .31, *p* < .001) The indicator of **anxiety symptoms** (BSI_Anx, *R*^2^ = .29; adj. *R*^2^ = .28) was predicted by = • Emotion-Oriented Coping (*β* = .490) • Low Identification (*β* = − .12) • Task-Oriented Coping (*β* = − .10)
19	Parker and Martin (2011)	200 clergy from Australia (Pentecostal, Churches of Christ, Presbyterian, Baptist, Greek Orthodox, Catholic, Anglican, and other) 78% men, 22% women Mean age 50 years old	Participants were contacted via mail or through the central body of their denomination, with return rates of approximately 35%,	The motivation and engagement scale-work (MES-W) (Martin 2007) = three adaptive cognitions of mastery, valuing and self-efficacy; the three adaptive behaviors of planning, persistence and task management; the three impeding cognitions of failure avoidance, anxiety and uncertain control; the maladaptive behaviors of self-handicapping and disengagement The Maslach Burnout Inventory was developed to assess the experience of burnout (Maslach, 1982): Emotional exhaustion EE, Depersonalization D and Personal Accomplishment PA Engagement and well-being: (Schaufeli et al. 2002): work satisfaction, workplace buoyancy, participation, positive future career plans	Correlational	Confirmatory factor analysis using LISREL = 18-factor model consisting of three burnout, four engagement and eleven motivation factors (*df* = 740; V2 = 1,413; RMSEA = .06; CFI = .96; NNFI = .95) The **success-oriented group, overstrivers** reported lower buoyancy (*β* = − .27, *p* < .001) and higher emotional exhaustion (*β* = .16, *p* < .01) T**he success-oriented group, self-protectors** reported lower levels of buoyancy (*β* = − .59, *p* < .001), participation (*β* = − .35, *p* < .001), enjoyment (*β* = − .43, *p* < .001), future career plans (*β* = − .42, *p* < .001), and personal accomplishment (*β* = − .46, *p* < .001) and higher levels of depersonalization (*β* = .51, *p* < .001) and emotional exhaustion (*β* = .38, *p* < .001) **The success-oriented group, failure acceptors** reported lower buoyancy (b = − .22, *p* < .001), participation (*β* = − .22, *p* < .001), enjoyment ((*β* = − .22, *p* < .001), future career plans ((*β* = − .15, *p* < .001) and personal accomplishment (*β* = − .19, *p* < .001) but were not significantly different on either depersonalization ((*β* = .02, ns) or emotional exhaustion (*β* = − .10, ns)
20	Raj and Dean (2005)	50 diocesan priests and 51 religious priests India Mean age 45 years old	Data were collected during the time they met for their monthly day of recollection. Data were collected by a personal visit to their respective communities within a two-week time frame	The Maslach Burnout Inventory was developed to assess the experience of burnout (Maslach, 1982): Emotional exhaustion EE, Depersonalization D and Personal Accomplishment PA The Center for Epidemiological Studies–Depression Scale (CES-D) The Self-Report Inventory (Virginia, 1998): age, the number of years in the priesthood, and number of clergy and religious brethren with whom participants currently live, vocational satisfaction, social support, spiritual life	Quasi-experimental design	Correlation between = **Vocational satisfaction and personal accomplishment** *r* = .29 and **depersonalization** *r* = − .34 were significant **Vocational satisfaction and depression** were significant, *r* = − .40, *p* < 05 **Social support and personal accomplishment** were significant, *r* = .29 **Relevant physical environment and personal accomplishment** *r* = .29, as well as Depersonalization *r* = − .22 were significant. All *p*_s_ > .05
21	Rossetti and Rhoades (2013)	2.482 Catholic priests (diocesan and religious) USA Mean age not provided. Mode was over 69 years old	Survey was mailed to every priest serving in 23 Roman Catholic dioceses. Varying sizes of dioceses responded, including five large archdioceses; four small, rural dioceses; and 14 moderate sized dioceses	The Maslach Burnout Inventory was developed to assess the experience of burnout (Maslach, 1982): Emotional exhaustion EE, Depersonalization D and Personal Accomplishment PA Measures spiritual practices = Variables = time off, good friends, childhood psychological problems, relationship to Bishop, inner peace, relationship with God	Correlational	**Burnout—Emotional Exhaustion** (*R*^2^ = .25**) Happy as priest = − 26*** Inner peace = 17*** **Burnout—Depersonalization** (*R*^2^ = 15***) Happy as priest = − .19*** Inner peace = − 18*** **Burnout—Personal Accomplishment** (*R*^2^ = .17***) Inner peace = .22*** Good friends = .16*** Relationship to God = .14***
22	Weaver et al. (2002)	Primary clergy (e.g., parish priest, lead pastor) representing the Roman Catholic, Methodist, Lutheran, and Baptist traditions). (*N* = 1288) Mean age 46 years old	Data collection = three steps: Searched electronic databases 1975–2000 to find published studies and mental health Examined the references of retrieved articles to identify additional research Consulted two experts in the field to identify any other studies	The research focused on three primary areas: morale and occupational stress, marital adjustment and family stress, and impairment (sexual misconduct)	Review of research	**Marital Adjustment and Family Stress** Greater loneliness, more emotional exhaustion, and lower marital adjustment, the lack of available social support, and intrusion on family life **Impairment (sexual misconduct)** Particularly vulnerable = Chronic stress; less confidence in their training as counselors
23	Webb and Chase (2019)	221 christian clergy (11.7% Catholic) USA Mean age 52 years old	Clergy were sent an email inviting them to complete a web-based questionnaire regarding their perceptions of the clergy vocation and its impact on their health Incentive = lottery to win one of two $100 gift cards	The International Physical Activity Questionnaire—Short Version (IPAQ-S) (Craig et al. 2003) The Clergy Occupational Distress Index (CODI) (Frenk et al. 2013) Health-related = list of chronic diseases Demographics = (years), sex, race, education level, years in ministry, hours worked per week	Correlational	Logistic regression predicting likelihood of reporting a **diagnosis of depression** Age *β* = − .04 *p* = .028* Sex *β* = − 1.41 *p* = . 002* Body Mass Index *β* = .01 *p* = .64 Physical activity *β* = .00 *p* = .38 Occupational distress. *β* = .15 *p* = .024*
24	Wells (2013)	883 sole or senior pastors (majority of respondents being Protestant or Catholic). First-part series of the study USA Mean age not provided. 54% are 51 years old or older	Telephone interviews	The Clergy Survey Dataset (Ware et al. 1994) = emotional health and physical health Work-related stress boundary-related stress (Wells 2010)	Correlational	Linear Regression = Relationship between **work stress and boundary stress on physical health and emotional stress** **Work stress** $$\Leftrightarrow$$ *β* = .06, Physical health $$\Leftrightarrow$$Emotional health *β* = .18, **Boundary stress**$$\Leftrightarrow$$ *β* = .15, Physical health $$\Leftrightarrow$$Emotional health *β* = .22, **Age** 45–50. $$\Leftrightarrow$$ *β* = .13, Physical health $$\Leftrightarrow$$Emotional health *β* = .15 = − .09, 51–60. $$\Leftrightarrow$$ *β* = .18, Physical health $$\Leftrightarrow$$Emotional health *β* = − .089, 61 + $$\Leftrightarrow$$*β* = .26, Physical health $$\Leftrightarrow$$Emotional health *β* = .01, **Children (Ref: “yes”)** $$\Leftrightarrow$$ *β* = − .05, Physical health $$\Leftrightarrow$$Emotional health *β* = − .10, **Less than a bachelor’s**$$\Leftrightarrow$$ *β* = − .09, Physical health $$\Leftrightarrow$$Emotional health *β* = .02, **Obese**$$\Leftrightarrow$$ *β* = .15, Physical health $$\Leftrightarrow$$Emotional health *β* = .06, **Length of time in ministry** High $$\Leftrightarrow$$ *β* = .08, Physical health $$\Leftrightarrow$$Emotional health *β* = .05, Low$$\Leftrightarrow$$ *β* = .05, Physical health $$\Leftrightarrow$$Emotional health *β* = − .07. (All *p*s < .05)
25	Wells et al. (2012)	883 sole or senior pastors (majority of respondents being Protestant or Catholic). Second-part series of the study USA 93% men, 7% women Mean age not provided 74,2% of the sample was older than 45 years old	Telephone interviews	The Clergy Survey Dataset (Ware et al. 1994) = emotional health and physical health Work-related stress boundary-related stress (Wells 2010) Control variables = race, age, gender, education, theological training, BMI, number of children in the home, second career, length of time in ministry, and bi-vocational status	Correlational	Linear association between **work stress and emotional and physical health** (all *p* < .0001) **PHealth** $$\Leftrightarrow$$ Adjusted *R*^2^ = .22 • Black clergy = *β* .11 • Older clergy = *β* = .23 • With children at home *β* = − .13 • Less than a bachelor’s degree *β* = − .2.1 • Bi-vocational status *β* = .06 **Emotional Healt**h $$\Leftrightarrow$$ Adjusted *R*^2^: .29 • White clergy = *β* -.17 • With children at home *β* = − .10 (All *p*s < .05) **Multiple regression = boundary stress and work stress** **Boundary stress** $$\Leftrightarrow$$ Adjusted *R*^2^ = .43 • Black clergy = *β* .18 • Older clergy + 61 = *β* = .12 • Married = *β* = − .29 • With children at home *β* = − .16 • Gender (male) = *β* = − .29 • Less than a bachelor’s degree *β* = − .24 • Obese *β* = .15 • Length of time in ministry = • High *β* = .29 • Low *β* = .22 • Very high *β* = .28 • Bi-vocational status *β* = .18 (All *p*s < .05)
26	Zickar et al. (2008)	Roman Catholic priests from a medium sized diocese USA Mean age 58 years old	First, the initial survey was pilot tested Then, the survey was mailed to all priests in the diocese	The adapted role ambiguity and role conflict scales (Rizzo, House, and Lirtzman, 1970) The Multidimensional Support Scale (Winefield, Winefield, and Tiggemann, 1992) Organizational Commitment Questionnaire adapted to the experience of a priest (OCQ) (Mowday, Steers and Porter, 1979) The abridged Job Descriptive Index (JDI; Stanton et al., 2002) = satisfaction with work, pay and coworkers The Job in General (JIG) scale (Stanton et al., 2002) measures global job satisfaction	Correlational	Hierarchical regressions **Social support from family and friends** was not a significant main effect **Social support from the bishop** was a significant main effect predictor of organizational commitment The remaining social support sources were significant buffers for: **Job satisfaction** Parishioners Δ*R*^2^ = .05* Staff Δ*R*^2^ = .03* Fellow Priests Δ*R*^2^ = .03*

### Sociodemographic Variables

Following to Arksey and O’Malley (2005) suggestions. We are analyzing factors related to study populations and its relationships with some important results. The first variable to bear in mind is priests’ age. Some studies found that young priests perceive more pressure than older ones. One possible explanation for this is that age is associated with the ability to develop internal strategies for coping with stressful situations and life challenges; in other words, the older the priest, the more (and better) strategies he employs (Büssing et al., 2017). Similar results were reported also by Raj and Dean (2005), who found that the feelings of emotional exhaustion experienced by priests who had been in the priesthood for 10 years or less were stronger and more intense than those experienced by their older counterparts.

In another study conducted in England and Wales, 1,468 questionnaires were completed by Catholic priests (both religious and diocesan). The measurement instruments were the Maslach Burnout Inventory (modified form) and the Eysenck Personality Questionnaire. The results revealed that priests aged between 40 and 49 scored higher for emotional exhaustion and depersonalization, while scores for personal accomplishment hardly varied at all across the different age groups. From age 60 onwards, scores for emotional exhaustion and depersonalization decreased. One possible explanation is that older clergy have learned to “make peace” with their job and have developed strategies for identifying the signals of impending burnout in order to take steps to avoid it (Francis et al., 2004). Similar results were found by Frick et al. (2015) and Webb and Chase (2019), who reported that older priests, who have to cope with fewer external stressors, have greater life satisfaction and better perceived self-efficacy.

Nevertheless, older priests do have to cope with certain age-related stressors: the need to take better care of their health as it gradually deteriorates (which generates anxiety, particularly among those who live alone), changes in residence (some move to care homes), a reduction in their social and ecclesiastical relevance (they no longer occupy positions of responsibility), the loss of loved ones and more free time, among others. Moreover, due to the lack of priests, older clergy often continue to perform different tasks and duties until a very advanced age. Faced with this situation, elderly priests need both effective coping strategies and acceptance and flexibility in order to learn to live with their new situation without losing sight of their own personal goals (Man-Ging et al., 2018).

A study conducted with 499 German clergy aged 65 and over (the majority diocesan priests) found that most had a good coping style and were less at risk of anxiety, depression and psychosomatic symptoms than those with avoidant behavior and negative emotionality. Similarly, identifying with the role of priest and with the organization, and having a strong spiritual life were found to be important protective factors (Man-Ging et al., 2018).

In another study, Kane (2017) held semi-structured interviews with 18 retired Catholic priests. The results reported are consistent with those outlined above, namely that older priests were satisfied with their life and ministry, were beginning to experience more health-related problems and, although they were slowing down, were nevertheless still keen to continue learning. However, due to the current scarcity of priests, many had no choice but to continue collaborating very actively in the pastoral activities of the diocese and felt there was a lack of support from the hierarchy and from parishioners, which resulted in loneliness. Respondents continued with their self-care routines as a good preventive system and protective factor. This included physical exercise, a healthy diet, routine medical care, finding time for private prayer and engaging in relaxation exercises.

Alongside age, another variable is working environment: rural or urban. One study reported that priests working in urban areas feel a greater sense of personal accomplishment than those working in rural environments (Raj & Dean, 2005).

Sociodemographic variables of the studies included in this review may be consulted in Table 3.

**Table 3:** Sociodemographic and relevant data

No.	Authors and year	Participants	Age (*M* and *SD*)	Gender	Education	Other data
1	^1^Adams et al. (2017)	[−]−	[−]−	[−]−	[−]−	[−]−
2	Beebe (2007)	343 serving clerics or recently retired	Ranging from 28 to 80. *M* = 54.6; *SD* = 9.42	223 men, 66 women, 1 unspecified	Not reported	Primarily White (92.7%), the remainder of the respondents were Black (2.4%), Hispanic (2.1%), American Indian (1.0%), Asian (0.3%), and Other (1.4%), with 3 participants not indicating any ethnic identification The sample represented clergy serving United Methodist (40%), United Church of Christ (21.7%), Evangelical Lutheran Church in America (14.8%), Roman Catholic (9.0%), Christian Church (Disciples of Christ) (6.9%), and African Methodist Episcopal, American Baptist, Episcopal, Jewish, Presbyterian, and non-denominational congregations (less than 5% each)
3	Büssing et al. (2013)	425 clerics	Ranging from 40 to 60. *M* = 58. *SD* not provided	Not reported	High school education	95,6% live alone, only 6,4% live in community. 241 worked as parish priests, 30 as pastoral counselors, 116 already retired
4	Büssing et al. (2017)	3.824 Catholic Priests from Germany	Ranging from 45 and 55 *M* and *SD* not provided	Not reported	High school education	All the sample living in celibacy. 66% of the sample lived alone, 20% lived with others in the same household and 14% lived in community with other priests
5	Ferguson et al. (2015)	539 lead clergy	*M* = 53 and *SD* not provided	81% men, 19% women	Not reported	Mean working hours per week = 46,76. 7,50 mean years at current church. 95% were white priests. 41% evangelical protestant, 2% black protestant, 44% mainline protestant, 7% catholic, 2% Jewish, 5% other religious tradition
6	Francis and Crea (2015)	155 Catholic Priests	Ranging from 24 to 76. *M* = 46 (*SD* = 12.16)	Not reported	Not reported	63% were Italians, 37% from other countries. 56% were diocesan priests, 44% religious’ priests
7	Francis and Crea (2018)	156 Catholic Priests and religious sisters	Sister ranging from24 and 74. *M*_*sister*_ = 50.6 (*SD*_sister_ = 13.5) Priests ranging from *27 to 86. M*_priest_ = 55.8 (*SD*_priest_ = 15)	61 women and 95 men	33 non-graduates, 123 graduates	[−]−
8	Francis et al. (2017)	155 Catholic Priests	Ranging from 24 to 76 M = 46 (*SD* = 12.16)	Not reported	Not reported	63% were Italians and 37% were from other countries. 56% were diocesan priests and 44% religious priests
9	Francis et al. (2004)	1.482 Catholic Priests (England and Wales)	*M* and *SD* not provided. 2% under 30, 13% in their thirties, 20% in their forties, 25% in their fifties, 26% in their sixties, 12% were in their seventies, and 2% in their eighties	Not reported	Not reported	[−]−
10	Francis et al. (2007)	1.482 Catholic priests. (England and Wales)	*M* and *SD* not provided. 2% under 30, 13% in their thirties, 20% in their forties, 25% in their fifties, 26% in their sixties, 12% were in their seventies and 2% in their eighties	Not reported	Not reported	[−]−
11	Frick et al. (2016)	8.574 priests from Germany	8.574 Catholic pastoral professionals (48% priests). 75% men, 25% women. *M* and *SD* not provided	75% were men and 25% women. Parish expert workers were mostly women (78%), while priests and deacons were exclusively men (100%). Pastoral assistants were both men (54%) and women (46%)	Not reported	[−]−
12	Greene et al. (2017)	103 Catholic diocesan priests from the USA	Ranging from 35 to 82. *M* = 58 (*SD* = 11.26)	Not reported	Not reported	Of the 46 religious and 57 secular/diocesan clergy, 66 self-identified as heterosexual, 31 self-identified as gay, and 6 self-identified as bisexual Ethnic: racial identity was predominantly non-Hispanic White (86%), with 2% Asian American, 2% American Native or Alaskan Native, 3% Black or African American, 6% Hispanic or Latino/a, 1% other identified Present assignment included 88% who were positioned in parishes and 12% in schools
13	Isacco et al. (2014)	15 Catholic Priests from the USA	Ranging from 29 to 76. *M* = 47, *SD* not provided	Not reported	Not reported	Participants had a wide range of years in the priesthood (< 6 months to 50 years; *M* = 16.2 years)
14	Isacco et al. (2016)	15 Catholic priests from the USA	Ranging from 29 to 76. *M* = 47 *SD* not provided	Not reported	Not reported	All of the priests were White. Range of years in the priesthood from less than 6 months to 50 years (*M* = 16.2 years). Nine participants were pastors, 5 were parochial vicars and 1 participant did not report his role
15	Joseph et al. (2011)	511 Catholic priests from India	Ranging from 27 to 88*. M* = 43 (*SD* = 11.8)	Not reported	43.2% bachelor, 44.4% master, 11.5% Ph.D 4 not report the educational level	The participants’ ministerial experience varied from 1 to 58 years, with a mean of 14.9 years (*SD* = 11.9), 28.2% of the participants lived alone without a companion priest, 26.4% had 1 priest companion, 16.8% had 2, 8.4% had 3, and the remaining 20.2% lived with between 5 and 38 companion priests
16	Kane (2017)	18 Catholic priests from the USA	*M* and *SD* not provided. All priests above 45 years old	Not reported	Not reported	The mean number of years of experience as a priest was 40.4 years (minimum = 20; maximum = 54)
17	López et al. (2014)	881 priests from Mexico, Central America and the Caribbean	*M* = 46 (*SD* = 11.58)	Not reported	Not reported	[−]−
18	Man-Ging et al. (2018)	499 German Catholic priests from Germany	*M* and *SD* not provided. All priests above 65 years old	Not reported	Not reported	They were divided into two age groups (A = 65–74 and B = 75–85 years) were mostly German citizens. (95%) living alone (A = 56%; B = 41%). Considering the participants’ age, many of them had health concerns: hypertension problems (A = 52%; B = 54%), diabetes (A = 20%; B = 17.5%), obesity (A = 25%; B = 21.7%); about 55% regularly consumed alcohol, and 10% were smokers
19	Parker and Martin (2011)	*N* = 200 clergy (Australia):a (Pentecostal, Churches of Christ, Presbyterian, Baptist, Greek Orthodox, Catholic, Anglican, and other)	*M* = 50 (*SD* = 10.3)	78% men, 22% women	Not reported	The average years of experience was 14.8 (SD = 9.7)
20	Raj and Dean (2005)	*N* = 101; 50 diocesan priests and 51 religious priests from India	Range from 30 to 91. *M* = 45. *SD* not provided	Not reported	Not reported	35.4% of priests lived in rural areas
21	Rossetti and Rhoades (2013)	2.482 Catholic priests (diocesan and religious) from the USA	Mean age 63 years old. The age spread was as follows: age 25–29 years *N* = 24 (1.0%) age 30–39 years *N* = 169 (6.8%); age 40–49 years *N* = 352 (14.2%); age 50–59 years *N* = 522 (21.0%); age 60–69 years *N* = 680 (27.4%) and over 69 *N* = 726 (29.3%)	Not reported	Not reported	51 (2.1%) Hispanic, 34 (1.4%) African, 14 African American (0.6%), 2,251 Caucasian (90.7%), 19 (0.8%) Vietnamese, 14 (0.6%) Filipino, 45 (1.8%) Polish, and 23 (0.9%) from India: 20 (0.8%) participants checked “other,” and there were 11 (0.4%) missing responses
22	^1^Weaver et al. (2002)	[−]−	[−]−	[−]−	[−]−	[−]−
23	Webb and Chase (2019)	221 clergy	*M* = 51.9 (*SD* = 12.1)	164 men (74,2%), 57 women (25,6%)	206 (93,1%) Master	210 participants were white (95%)
24	Wells (2013)	883 sole or senior pastors (majority of respondents being Protestant or Catholic). First-part series of the study USA	*M* and *SD* not provided. 54% are 51 years old or older	93% men, 7% women	Not reported	Whites comprise the majority (82%), and African Americans make up 13% of the respondents. Seventy-three percent of the respondents are currently married, and 55% currently have no children at home. Fifty percent of the clergy report that they are overweight, and 27% report obesity
25	Wells et al. (2012)	883 sole or senior pastors (majority of respondents being Protestant or Catholic). Second-part series of the study USA	*M* and *SD* not provided. 74,2% of the sample was older than 45 years old	Among all participants, 93.0% are men and 7.0% are women. Among Catholics, 100% of the participants were men. Among mainline Protestants, 80.0% of the respondents were men. Ninety-nine percent of the conservative Protestants were men; ninety-seven percent of the historically African American respondents were men	Not reported	Whites comprised 82.0% of the respondents; African Americans comprised 13%; other races made up 4% of the sample. 621 (/70,3%) participants were married while 262 (29,7%) were not
26	Zickar et al. (2008)	190 Roman Catholic priests from a medium sized diocese from the USA	*M* = 57.9 *SD* not provided	Not reported	Not reported	[−]−

No data are provided for reviews of research (Adams et al., 2017; Weaver et al., 2002)

### Psychological Factors and Coping Styles

Following Scopus review suggestions, in this section we are analyzing the relationships between important factors on stress among priests. The personality characteristics that lead to burnout are mainly found in idealists, perfectionists and compulsives (Raj & Dean, 2005). In the studies analyzed, high scores for extraversion correlated with low levels of emotional exhaustion and depersonalization and high levels of personal accomplishment, whereas high scores for neuroticism correlated with high levels of emotional exhaustion and depersonalization and weak feelings of personal accomplishment (which in turn are linked to low expectations regarding self-efficacy). Finally, high scores for psychoticism were associated high levels of emotional exhaustion and depersonalization (Francis et al., 2004).

In a study conducted with 155 Catholic priests (63% Italian and 37% from other countries; 56% diocesan and 44% religious), work-related health was analyzed in relation to psychological type: extravert or introvert. The principal conclusion drawn was that introverted priests have poorer work-related psychological heath than extraverted ones. This is mainly manifested in their poor results for emotional exhaustion and satisfaction with their ministry. The priesthood seems to attract more introverts than extraverts, since 59% of participants in the study fell into this psychological type. Nevertheless, the priestly role involves a great deal of public presence and intense interpersonal demands and requires clergy to interact with people in different contexts—something which seems much more suited to an extraverted personality (Francis & Crea, 2015). Nevertheless, introversion fosters a deeper spiritual life, which is very important to a priest.

Francis and Crea (2018) also explored the link between personality, happiness and psychological health among Italian priests (*n* = 95) and nuns (*n* = 61) (studied together in a single sample). The results support previous hypotheses: extraversion predicts high scores for happiness and satisfaction with ministry and low scores for emotional exhaustion. For its part, happiness (a concept taken from positive psychology) also serves as a predictor of psychological health (positive emotionality), although it is a concept that is constantly being redefined.

In a study carried out with 511 Indian Catholic diocesan priests, Joseph et al. (2011) linked the three subscales of burnout syndrome with personality factors in accordance with the Big Five and engagement. The results revealed significant (*p* < 0.01) positive correlations between neuroticism and emotional exhaustion (*r* = 0.53) and between neuroticism and depersonalization (*r* = 0.50), and negative correlations between neuroticism and personal accomplishment (*r* = − 0.44) and between neuroticism and engagement (*r* = − 0.50). In contrast, extraversion correlated positively with personal accomplishment (*r* = 0.33) and engagement (*r* = 0.33) and negatively with emotional exhaustion (*r* = − 0.34) and depersonalization (*r* = − 0.32). Similar results were found for agreeableness and conscientiousness, with results revealing negative correlations between agreeableness and emotional exhaustion (*r* = − 0.51) and between agreeableness and depersonalization (*r* = − 0.56) and positive correlations between conscientiousness and personal accomplishment (*r* = 0.35) and between conscientiousness and engagement (*r* = 0.44). No statistically significant correlations were observed between openness to experience, and the other variables studied.

Another key aspect to take into consideration is the way in which priests manage their emotions. In a study exploring how positive and negative emotions affect stress, Francis et al. (2004) found that the pressure of working with people all day results in around 25% of priests feeling exhausted, fatigued when they get up in the morning, weighed down by their responsibilities (emotionally drained) and blamed by parishioners for their problems, all of which renders them less patient, less able to listen and less concerned about what happens to their flock (depersonalization). Nevertheless, this negative emotionality is balanced by feelings of personal satisfaction at working with people (90%) and by the conviction that what they do has a real positive impact on people's lives (70%). It is also true, however, that only one-third of priests consider themselves to be truly effective in their job (32%) and describe themselves as being very energetic (31%).

With the same sample of 155 Italian Catholic priests described earlier in this paper, the same authors found that negative emotionality was mainly manifested in a cynical and biting sense of humor (20%), a feeling of discouragement due to lack of support (23%) and daily feelings of fatigue and irritation (28%). Nevertheless, it was positive emotionality that was dominant in participating priests, manifested as a feeling of having made many worthwhile accomplishments in their current ministry (77%), the conviction that their ministry gave real meaning and purpose to their lives (83%) and the fact that they felt very happy with their decision to join the priesthood (90%) (Francis et al., 2017). Similar results were reported by a study conducted by Francis and Crea (2018) 1 year later.

It is also important to take narcissistic styles within the clergy into account. The “messiah complex and mentality” means that both the priest himself and those who surround him have extremely high expectations of his performance. A priest who tries to serve a community to the very best of his ability and who sees it as his duty to be available 24 h a day, 7 days a week, will feel guilty about setting limits or turning down a request. A priest with narcissistic tendencies, which prompt him to constantly prove his staunch commitment and dedication, will struggle with high levels of stress and negative emotions (Isacco et al., 2014). Priests often feel the weight of their role and the pressure to always convey an appropriate image. In general, people join the priesthood with high ideals, enormous optimism and a strong commitment to helping people. However, as difficulties accumulate, priests may become susceptible to feelings of disillusionment and despair (Raj & Dean, 2005).

Another factor linked to coping styles is expectation of self-efficacy, which is a protective factor against perceived stress (Frick et al., 2015). Büssing et al. (2013) found a statistically significant negative correlation between perceptions of self-efficacy and spiritual dryness associated with burnout syndrome among clergy (*r* = − 0.305; *p* < 0.01). Priests with good self-efficacy expectations feel confident that they can successfully cope with unexpected events and find different solutions to problems. They also feel that if someone opposes them, they can find the means and ways to get what they want.

This need for differentiation has prompted some authors to suggest that authenticity (beyond the priestly role) and being constantly reminded of one’s individuality may be protective factors (Isacco et al., 2014).

Moreover, according to Zickar et al. (2008), role ambiguity, role conflict and role overload correlate negatively with job satisfaction and organizational commitment (to the diocese), whereas role ambiguity correlates negatively with (mainly) job satisfaction (*r* = − 0.41; *p* < 0.001) and organizational commitment (*r* = − 0.23; *p* < 0.001).

### Living Conditions

Another interesting category found in our research is related to the priest’s living conditions. Specifically, one basic aspect of priests’ living conditions is their place of residence. In many cases, since their living quarters adjoin their place of work, priests are available round the clock, being obliged to accede to all demands and attend to all requests, never being able to disconnect from their job (Isacco et al., 2014). Indeed, the limits between clergy’s public and private life often become blurred (Frick et al., 2015). Furthermore, according to Raj and Dean (2005), the possibility of having a positive physical environment has a significant impact on personal accomplishment (*r* = 0.29, *p* < 0.05) and depersonalization (*r* = − 0.22, *p* < 0.05).

Another aspect is loneliness, which particularly affects diocesan priests who often live and work alone. Loneliness has frequently been associated with major symptoms of depression and burnout (Greene et al., 2017; Raj & Dean, 2005).

In relation to how having hobbies and engaging in community life help minimize the negative impact of burnout among priests, findings indicate that while they may both be protective factors, the results fail to reach statistical significance (Francis et al., 2017).

Over recent years, studies have shown how having a pet can benefit people’s occupational health. In a sample of 1,468 Catholic priests from England and Wales, it was found that 11% of clergy had a dog and 6% had a cat. Although previous research has found important social, psychological and medical benefits to owning a pet, no statistically significant association has been reported between having a cat and the three dimensions of burnout (emotional exhaustion, depersonalization and personal accomplishment). Moreover, among dog owners, scores for emotional exhaustion and depersonalization increased slightly, while scores for personal accomplishment were unaffected. One conclusion drawn by authors studying the clergy is that the results obtained with other populations can rarely be extrapolated to the situation experienced by priests. Thus, while owning a pet seems to facilitate social relations among people who feel isolated, in the case of priests, these interactions already exist, and are indeed plentiful, which is perhaps why the benefits of pets have not been observed among this group. Moreover, the fact of having to cope with illness, death and suffering on a daily basis prompts clergy to develop resources for coping with their own vulnerability. Finally, given their generally very busy schedules, priests may view pets more as just another responsibility (particularly in the case of dog owners) than as a source of “companionship” (Francis et al., 2007).

### Sociocultural Context

We have found significant influence of sociocultural context in the reviewed papers. Another source of stress for members of the clergy is the negative image of the priesthood portrayed in the media, along with people’s negative reactions and attitudes of suspicion and mistrust toward clerics. Priests feel shame and sadness over the harm caused to victims by fellow members of the Church (Isacco et al., 2014).

Moreover, priests represent sacred values, such as celibacy, which are consistent with centuries of church tradition. However, due to today’s secularized society, fewer and fewer people are called to the priesthood, which in turn generates overload and concern about the future (Rossetti & Rhoades, 2013). According to Kane (2017), in the USA, the number of Catholic priests has dropped over the last 50 years (1965–2014) from 58.632 to 38,275. Five decades ago, 94% of priests were involved in active ministry, whereas today, this number is just 68%. Nevertheless, the number of parishes has remained the same, meaning that congregations that were previously served by more than one member of the clergy today have only one priest who, moreover, often has to divide his time between various different parishes.

### Demands of the Job

As current research show, and based on demands-resources model, we found the following results. As regards the demands to which priests are subject, the list provided by authors seems endless. Firstly, clergy often have the feeling that their job never ends. According to some studies, many of them work up to 63 h per week and feel pressured into engaging in multiple activities, leaving them little free time for themselves. Moreover, they often perform highly repetitive tasks and work for many years with the same people (Raj & Dean, 2005; Rossetti & Rhoades, 2013).

Secondly, priests cannot always measure their job in terms of efficacy since there is often a lack of tangible results. Moreover, it is frequently hard to determine right from wrong in the decisions they make, leaving them vulnerable to criticism (Adams et al., 2017; Rossetti & Rhoades, 2013).

Some specific tasks are particularly stressful; for example, officiating at funerals, helping parishioners cope with grief (Adams et al., 2017), and social-charitable work (Rossetti & Rhoades, 2013). Being counselors is also sometimes difficult and priests need sufficient psychological tools to ensure that they can give good help and advice (Kane, 2017; Weaver et al., 2002). In many cases, tasks such as serving various different parishes or administering sacraments can only be carried out by priests, who consequently feel overwhelmed (Isacco et al., 2014).

Organizational, management and administrative tasks are often perceived as stressful, since they are very time-consuming and not particularly appreciated by parishioners (Raj & Dean, 2005). Furthermore, in relation to tasks such as doing the parish accounts, for example, priests are rarely trained or qualified for this job yet spend an enormous amount of their time doing it (Isacco et al., 2014).

The priestly lifestyle involves juggling multiple roles and boundaries and managing conflicts in the parish. Interpersonal boundaries can often be ambiguous. Working with people is a vital part of the priestly vocation and can be simultaneously both satisfactory and stressful. Priests sometimes think that people “do not hear the word of God,” constantly complain about parish activities and are an endless source of conflict. It is certainly true that each individual has different priorities and a different history, and all personalities are complex. At the same time, however, and although managing multiple leaderships within a community is a complex task, priests acknowledge that those who do participate in parish life have much of value to contribute (Isacco et al., 2014).

Finally, dealing with people’s expectations regarding what it means to be a good priest is often another source of stress and tension (Beebe, 2007; Isacco et al., 2014; Raj & Dean, 2005; Rossetti & Rhoades, 2013).

### Organizational Variables

Related to organizational variables, we found some interesting results as well. The priests participating in the studies reviewed often referred to a lack of social support by parishioners and collaborators (feelings of isolation and loneliness), as well as to the absence of “networking” with other colleagues, as factors which increased their occupational stress (Büssing et al., 2017). Indeed, Zickar et al. (2008) found significant associations (*p* < 0.001) between support from fellow priests and parish staff and commitment to the diocese and job satisfaction. Thus, support from parish staff correlated positively with commitment to the diocese (*r* = 0.20) and job satisfaction (*r* = 0.29). For its part, support from fellow priests also correlated positively with commitment to the diocese (*r* = 0.32) and job satisfaction (*r* = 0.18).

These results are consistent with those found by Raj and Dean (2005). According to their study, social support has a positive impact on priests’ sense of personal accomplishment (*r* = 0.29, *p* < 0.05).

One key organizational variable is the attitude of bishops toward the clergy under them. Bishops are often remote and unapproachable figures, exercising their authority in such a way as to ensure an intensely hierarchical organizational system. In the Catholic Church, bishops determine whether or not deacons can be ordained, and once they are, to which parish they will be assigned, where they will live and how long they will have to work before retiring, etc. (Kane, 2017; Raj & Dean, 2005). Returning to the study by Zickar et al. (2008), support from the bishop correlated strongly with commitment to the diocese (*r* = 0.57; *p* < 0.001).

As regards priests’ assessment of bishops as leaders of their organization, Kane (2017) points out that, in some recent studies, 68% of clergy report a low level of trust in their bishop and his skills as a leader of diocesan pastoral life. At the same time, 59% of participants claimed that they receive minimal (or no) support from their bishop in response to excessive or unrealistic demands from parishioners that go well beyond both their abilities and availability. Bishops are not able to demonstrate sufficient levels of respectful leadership; they do not take the needs of their collaborators into account; they have trouble fostering an organizational climate conducive to personal growth; and are incapable of reconsidering a decision in light of disagreement from priests. The author concludes that bishops seem to be overly concerned with defending their power, even when this gives rise to unfair situations.

### Vocational and Spiritual Variables

Another group of stressors is linked to the priestly role and vows of obedience. Specifically, the vow of obedience generates most difficulties when it is understood as blind, submissive following of orders and when, for example, it involves accepting a transfer to a different parish, with the personal and relational uprooting that this implies (Isacco et al., 2014).

In a qualitative study carried out with 15 diocesan priests from the USA, participants claimed that their promise of obedience had a positive effect on their health since it increased positive feelings linked to a freely made commitment, provided stability and fostered a stronger relationship with the Church. However, as explained above, obedience sometimes generates negative emotions, especially when priests come into conflict with their bishop. Celibacy also generates positive feelings. Moreover, by not being committed to one single person, priests can enjoy a varied and enriching set of relationships and are free to focus more on their vocation.

Büssing et al. (2013) explored spiritual dryness among Catholic priests in relation to burnout syndrome. Statistically significant (*p* =  < 0.01) negative correlations were observed between spiritual dryness and “positive” variables such as daily spiritual experiences (*r* = − 0.660), active religious practices (*r* = − 0.453), optimism (*r* = − 0.414), life satisfaction (*r* = − 0.434) and engagement (*r* = − 0.438). Moreover, as expected, spiritual dryness was also found to be negatively associated (positive correlations) with the three subscales of burnout: emotional exhaustion (*r* = 0.464), depersonalization (*r* = 0.450) and low personal accomplishment (*r* = 0.441). Finally, it is often accompanied by certain pathologies, such as anxiety (*r* = 0.387) and depression (*r* = 0.544).

In a subsequent study, Büssing et al. (2017) confirmed that the elements that best explain spiritual dryness are: a lack of perception of the transcendent, low sense of coherence (understood as being “anchored” in a meaningful life, with clear goals), depressive symptoms and emotional exhaustion (typical of burnout).

It should not be forgotten that since priests tend to attribute a sacredness to their work, they often work longer and harder than people in other professions in order to maintain an adequate image of themselves and to avoid perceived failure (Adams et al., 2017).

Prayer was also revealed as an important protective factor. Indeed, problems with stress are sometimes linked to having given up the habit of praying. Prayer is associated with spiritual well-being (Isacco et al., 2014), and some authors have reported that the more time priests spend praying, the lower their level of emotional exhaustion. Nevertheless, nothing in the results suggests that praying has a positive impact on satisfaction in ministry (Francis et al., 2017), although those who spend more time engaged in spiritual reading and prayer were found to have a stronger sense of personal accomplishment and fewer symptoms of depression and burnout (Raj & Dean, 2005).

Priests’ relationship with God is also mentioned in some studies as a protective factor. Clergy who feel a connection with God are better able to deal with important issues, feel themselves to be an instrument in God’s hands and view God as a traveling companion who supports them and gives meaning to their lives. Faith in God is experienced as a dynamic relationship which is intrinsically linked to their maturity as people. It also provides a connection which fosters positive emotions, decreases the impact of negative emotions and enables authenticity (Isacco et al., 2016).

Having a purpose in life correlates positively with satisfaction in ministry, and both elements correlate negatively with emotional exhaustion (Francis et al., 2017). Similar results were reported also by Raj and Dean (2005), who found significant correlations between vocational satisfaction and personal accomplishment (*r* = 0.29, *p* < 0.05) and between vocational satisfaction and depersonalization (*r* =  − 0.34, *p* < 0.05). Personal accomplishment was also fostered by spiritual activities (*r* = 0.21, *p* < 0.05).

### Comparative Studies

Some studies present a sample of Christian ministers, including Catholic priests, highlighting those aspects that all have in common. For instance, in a sample of 1,288 clergy from different Christian denominations (Roman Catholic, Lutheran, Methodist and Baptist) in the USA, the authors found strong correlations between high scores for occupational stress and high blood pressure (33.5%), high cholesterol (29%), type 2 diabetes (10.4%), heart disease (6.8%), lung disease (2.3%), arthritis (15.4%), chronic stress disorder (0.5%), hours worked per week (mean of 46.1 h) and a sedentary lifestyle (mean of 6 h sitting a day) (Webb & Chase, 2019). Other problems associated with work-related stress are: increased anxiety and depression among priests, isolation, sleep disorders, medical issues and feeling angry all the time (Greene et al., 2017; Webb & Chase, 2019).

In the USA, 883 clergy from different Christian denominations responded to a questionnaire in which work-related stressors (conflicts, criticism about their ministry, role ambiguity, congregational challenges, etc.) were linked to boundary-related stressors (congregational demands, isolation, lack of personal and private time, little time for recreation). The results revealed a strong correlation (*r* = 0.63; *p* < 0.0001) and a linear relationship between the two constructs (Beta = 0.81, SE = 0.03; *p* < 0.0001), with multiple regression analyses indicating that as work-related stress increases, boundary-related stress also becomes more intense (Wells et al., 2012).

As regards coping styles, Parker and Martin (2011) carried out a study with 200 Australian clergy from eight different Christian denominations (including the Catholic Church). The authors measured two basic job-related behaviors: approach (hope for success) and avoidance (fear of failure) and established four coping styles linked to participants’ self-assessments: “success oriented” (high approach/low avoidance), “overstriving” (high approach/high avoidance), “self-protecting” (low approach/high avoidance) and “failure accepting” (low approach/high avoidance). Success-oriented clergy were found to be more engaged in their work, to use better cognitive and behavioral strategies (mastery orientation and self-efficacy, task management, planning and persistence, etc.) and to have lower burnout rates. Self-protecting clergy, on the other hand (who had higher levels of anxiety, lower levels of mastery orientation and poorer capacities), scored lower on both scales. The overstriving group scored highly for emotional exhaustion and the failure accepting group scored very low for engagement.

We have also seen that physical activity is an excellent preventive measure. Webb and Bopp (2016) designed a study with two groups of Christian clergies. One group followed a three-month physical activity regime specially designed for clergy called “Walking in faith,” whereas the other “control” group followed no specific treatment. Clergy from both groups were selected on the basis of having a sedentary, insufficiently active lifestyle, being at an age characterized by intense pastoral activity and not having problems preventing them from engaging in physical exercise. The 12-week regime linked biblical texts with health recommendations, warning about the risks of a sedentary lifestyle, promoting the value of an active life, highlighting the importance of seeking social support when engaging in physical activity and explaining how physical activity can help people cope better with stress, etc. Biblical references were used to adapt the regime to participants’ culture and beliefs (proven to be much more effective than the use of a standard program). As expected, the results revealed significant improvements in the sedentary behavior, physical activity and perceived self-efficacy of those in the experimental group, in comparison with the controls.

As to the differentiation of self and role, in a study in the USA with 343 clergy from different Christian denominations (Catholic priests represented 9% of all participants), Beebe (2007) found that when individuals feel able to distinguish between their personal identity (self) and their priestly role, as two clearly differentiated realities, they do not tend to over-identify with the congregations they serve (fusion) and do not experience a feeling of overload in relation to the demands placed on them. The ability to differentiate between self and role was found to predict low emotional exhaustion, low depersonalization and high personal accomplishment. For their part, role ambiguity and overload were associated with all three burnout symptom scales. High self-role differentiation was also linked both to better conflict management and to the competitive (particularly useful in emergency situations or when group values are at stake) and collaborative (searching for a mutually satisfying solution that takes everyone’s interests into account) conflict resolution styles, whereas poor self-role differentiation was associated with the avoiding and accommodating conflict resolution styles.

According to the effort-reward imbalance model, the priesthood could be included in the helping professions category. All helping professions involve high levels of intrinsic (perfectionism, job commitment, difficulty disconnecting from work obligations and a pressing need for success and approval) and extrinsic effort (heavy workload, scarce resources, high level of responsibility and role conflicts), coupled with low rewards (difficulties bringing about change in the world, earning a good salary, gaining other people’s approval, developing one’s professional career, etc.). Comparing the priesthood with other professions may help gauge the degree of work-related stress and burnout to which clergy are subject (Adams et al., 2017).

In a review of the literature (84 studies), Adams et al., 2017 found similar levels of emotional exhaustion among clergy from different Christian denominations as among social workers, counselors and emergency personnel, and lower levels in comparison with teachers and police officers. However, clergy were found to have higher levels of depersonalization than social workers and counselors, similar levels to teachers and lower levels than emergency personnel and police officers. Finally, priests had moderate burnout levels in terms of personal accomplishment, with their scores being higher than those of counselors, similar to those of social workers and teachers and lower than those of police officers and emergency personnel. Given the high stress levels to which clergy are subject, their moderate burnout levels suggest that they employ good coping strategies.

Using a similar approach, Rossetti and Rhoades (2013) administered a detailed questionnaire to a sample of 2,482 Catholic priests in the USA. Clergy were found to score lower for burnout than both the general population and those in other professions considered particularly vulnerable in this respect, such as medicine or the social services. Some of the elements found to protect priests from burnout were a satisfying spiritual life and a sense of inner peace, being happy as priests and satisfied with their job, regular physical exercise, having good friendships, having a good relationship with and feeling supported by colleagues and their bishop, having one day off a week and having a good self-image.

Other studies have focused on the differences between Catholic priests and clergy from other religions. For example, in comparison with Anglican clergy, Catholic priests from Wales and England were found to have higher levels of emotional exhaustion and depersonalization, although they also had a greater sense of personal accomplishment (Francis et al., 2004).

This group of studies also includes a literature review by Weaver et al. (2002), in which the authors found that Protestant clergy reported higher mean levels of occupational stress, stating that they felt isolated, unsupported, vulnerable and angry with parishioners, and that they had difficulty establishing boundaries. Catholic priests, brothers and sisters, on the other hand, reported fewer vocational pressures and claimed to feel more supported by their colleagues and institutions. Finally, the fact that Protestant clergy can marry was found to be positively linked to marital commitment, satisfaction and good communication. However, the level of public exposure to which the families of Protestant clergy are subject and the pressing demands of the job, which make striking a good work-life balance difficult, are important stressors which, on occasions, result in clergy leaving the ministry.

Comparisons can also be made within the Catholic priesthood between diocesan priests and priests belonging to religious orders (or religious priests). To this end, Raj and Dean (2005) conducted a study with Catholic priests in Southern India (50 diocesan priests and 51 religious priests), administering the Maslach Burnout Inventory and the Center for Epidemiological Studies–Depression questionnaire. Diocesan priests obtained worse results than their counterparts in religious orders in all three subscales of the Maslach Burnout Inventory (emotional exhaustion, depersonalization and personal accomplishment) and reported more depressive symptoms (*M*_diocesan_ = 1.91 vs. *M*_religious_ = 1.60). Although no significant differences were observed between the two groups in either vocational satisfaction or spiritual activities, diocesan priests did score lower for social support (*M* = 18.96 vs. *M* = 21.72) and relevance of the physical environment (*M* = 10.67 vs. *M* = 11.66). The authors also observed differences in proactive behaviors within the organization. Thus, while diocesan priests were not encouraged to specialize in a field that may awaken their interest and felt themselves to be intensely involved at a personal level with the problems of their parishioners, religious priests tended to spend most of their time engaged in “non-pastoral” activities such as social work or educating children and youths, and felt encouraged by their superiors to further their training in those areas of knowledge best suited to their individual profile and the challenges faced by the religious order to which they belonged.

Within the Catholic Church, comparisons can also be made between the different pastoral professions in terms of the stress to which each is subject. For instance, in a study conducted in Germany with 8.574 pastoral professionals, of which 4.157 were priests, it was found that men had a worse perception of work-related stress, lower expectations of self-efficacy and lower levels of life satisfaction. The high scores reported in general for men (75.1% of the total sample) were strongly influenced by the results corresponding to priests (48% of the total), who scored highest for depression, somatization, stress perception and spiritual dryness and lowest for self-efficacy expectations and life satisfaction (Frick et al., 2015).

### Stress, Burnout and Health

The last category included in our scoping review is the relation between stress, burnout and health. In a study designed to analyze how work-related stress and burnout affect clergy’s health, Ferguson et al. (2015) found a significant relationship between stress and obesity. Clergy who feel more pressured, criticized, isolated and “bi-vocational” (i.e., feel they have more than one job) tend to experience greater stress and are more likely to become obese. Priests who are stressed see themselves as having fewer coping resources and their response is “evolutionary”: they eat more fat and carbohydrates to ensure their “physical survival.” Moreover, stress discourages physical exercise and may even alter the metabolism.

Similar results were reported by Wells (2013), who linked work-related stress and stress associated with a lack of interpersonal boundaries using participants’ Body Mass Index. The results revealed a positive correlation between work-related stress and obesity (*r* = 0.18; *p* = 0.0001) and a slightly stronger one between boundary-related stress and obesity (*r* = 0.21; *p* = 0.0001).

In a study with 881 priests from Latin America, the authors found that 39.62% of participants had no symptoms of burnout, 60.38% had moderate levels of this disorder in some or all of its three dimensions, and 25.39% presented the syndrome in its fullest extent, with emotional exhaustion being the factor that best explained the problem (26.22% of participants). A statistically significant correlation was observed between emotional exhaustion and smoking (*r* = 0.365; *p* = 0.007), and depersonalization correlated with both alcohol use (*r* = 0.378; *p* = 0.001) and smoking (*r* = 0.333; *p* =  < 0.001). However, in this study at least, personal accomplishment did not seem to affect the manifestation of burnout among clergy (López et al., 2014).

As protective factors, those who have one day off a week, take holidays, have the opportunity to take a sabbatical and have support groups (fellow clergy, friends, etc.) are less likely to suffer from stress. Having days off and rest periods enables clergy to withdraw from their public role, engage in physical exercise, cook healthy meals without being in a rush and spend time with their relatives. Physical exercise is one of the best predictors of occupational health. Also, support groups with peers are particularly healthy, since they give clergy the chance to share concerns and needs with people who have similar experiences in a safe, confidential environment, thus helping to mitigate the common feeling of social isolation (Ferguson et al., 2015; Webb & Chase, 2019).

In the “Results” section, four tables are presented to organize the results of our revision. First table summarizes the steps of a scoping review process (see Table 1). Table 2 shows the main findings of the studies included in this review. On the other hand, Table 3 summarizes the most important sociodemographic data which comprises the samples of the studies reviewed. Finally, Table 4 presents the results regarding risk and protective factors.

**Table 4:** Risk and protection factors for stress among Catholic priests

Risk factors for stress	Protection factors for stress
*Personal characteristics*	
Neuroticism	Extroversion, responsibility and friendliness
Perfectionism	Engagement and self-efficacy
External locus of control	Positive emotions (optimism)
Type A personality profile; Narcissism	Active coping style
Role orientation	Personal identity and authenticity
Avoidance coping style	Competitive and collaborative styles of conflict resolution
Over complacency	
*Life conditions*	
Mismanagement of loneliness	Possibility of finding rest periods: vacation, days off, daily rest
Absence of support network and friends	Physical exercise, healthy diet
Loneliness	Health check
Sedentary lifestyle	Relational world: other priests, friends, parishioners
Absence of rest periods	Spiritual life (personal prayer)
*Ecclesial and social context*	
Religious indifference	Seeing the current context as an opportunity for improvement and innovation
Spillage of social relevance	Learning to set adequate boundaries
Excessive demands and expectations of parishioners. Clergies should be always available	
*Organizational climate and job satisfaction*	
Ambiguity and role conflict	Clear terms of what is expected of clergy, better-defined tasks
Authoritarian leadership style	Leadership based on influence: motivating work climate
Pyramid scheme of work	Teamwork and networking
Lack of closeness and concern of diocesan authorities	Better opportunities to develop their skills (entrepreneurship, job crafting)
Blind obedience	

## Conclusions

As Arksey and O’Malley (2005) suggested, we followed the recommended steps to perform a scoping review of occupational stress factors over Catholic priest. We found some interesting results that may help readers to approach this complex topic. We can conclude that our objectives have been accomplished. We conducted a review of the most important factors found in the literature related to work-related Catholic priests’ stress. Besides, we have offered to the readers a wide categorized optic of the influence of diverse variables over work-related Catholic priests’ stress. Consequently, we can affirm that we have accomplished objectives 1 and 2 purposed in the current research. Having said this, we can conclude that catholic diocesan clergy, who live alone, have higher levels of anxiety and depression than those belonging to religious orders, who live within a community (Knox et al., 2002). According to Seghedoni (2012), rather than talking about loneliness or solitude, we should really be talking about “solitude*s*,” in plural, since affective solitude (partly foreseen by clergy) is accompanied by pastoral solitude and institutional solitude also, which are often more harmful. According to this author, encounters between priests are often characterized by haste, pressure to make headway in a joint project and “gossip.”

As seen earlier in this chapter, support from fellow priests is a powerful protective factor against stress (Zickar et al., 2008). For Uriarte (2010), a sense of “fraternity” encourages a spirit of collaboration among clergy, which in turn helps them be more efficient and avoid the loneliness of a “long-distance runner.” However, as this author claims, fraternity is, above all, a question of identity: “Priests need other priests in order to be priests.” This is consistent with the principles of social identity, which argue that a large part of a person’s identity is constructed on the intergroup plane (Haslam et al., 2011).

Although the loneliness experienced by Catholic diocesan priests correlates strongly with mental health issues such as depression and stress, we should not idealize the family life of clergy from other denominations (Guzman and Teh 2016; Kim et al. 2016; Wilson and Darling 2017).

Turning from loneliness to the impact of age on stress, it is important to note that younger and middle-aged priests report higher stress levels. Among younger clergymen, this may, as Parolari (2006) suggests, be the result of their “insertion” into the priestly role. Young priests tend to focus on responding to the most obvious and pressing expectations in their environment (as expressed by people, the institution, their bishop, etc.), often without realizing the price that this will exact. They strive to earn the minimum level of consideration and esteem that provides them with confirmation of their own identity.

The fact that middle-aged priests also report high levels of work-related stress is consistent with that suggested by some authors, who argue that it is during this time in their lives that clergy are most productive and “generative.” It should also be remembered that many priests continue to work actively until the age of 75 or 80, and the results suggest that older clergy, who have fewer demands placed on them by an intense work schedule, are able to organize their working day in a more satisfactory manner (Garrido, 1997; Uriarte, 2011).

Priestly role also seems to play a key role in experiences of stress, with those who become absorbed in their clerical duties being more vulnerable to emotional exhaustion and depersonalization (Beebe, 2007; Zickar et al., 2008). According to Guarinelli (2014), we belong to a culture in which insistence on the search for one’s psychological identity can be very strong. Yet at the same time, priests’ psychosocial role no longer has the same force of identity as it did just a few decades ago. Perhaps this is why there is so much insistence on maintaining or recovering certain symbols, as a means of compensating for this weakening of the priestly identity.

Indeed, role ambiguity is an important explanatory factor for job dissatisfaction among clergy. Questions about role ambiguity referred to the clarity of the institution’s aims, the balanced distribution of working times, a clear explanation of what clergy were expected to do, a clear definition of responsibilities and specific knowledge of what was expected of them. When role ambiguity is low, job satisfaction is adequate even if role conflict is high (Faucett et al., 2013).

In this sense, Rulla () points out that the vocational role should be understood as a resource at the service of “internal vocational consistency,” rather than as an end in itself. Thus, the actions deployed within the role should serve to strengthen the internal system of attitudes and vocational values; in other words, behaviors associated with the role should be internalized. In this way, the role is oriented toward values, since it enables ideal modes of behavior; the role is chosen in accordance with the system of values, but said system transcends the role itself.

As seen earlier, the figure of the bishop is perceived as distant and incapable of engaging in effective leadership. In this sense, Kane (2014) observed that although bishops “in general” were negatively assessed in terms of their leadership skills, when speaking of their particular bishop, participants gave a more detailed opinion and rated them slightly higher. Nevertheless, the research to which the author refers reveals a consistent relationship between poor leadership by bishops and high levels of discouragement, distress and depression among priests, whereas, as seen in the introductory chapter (Haslam et al., 2011; Moriano et al., 2014; Serrano-Orellana & Portalanza, 2015), close and respectful leadership based on the key elements of social identity theory fosters (principally) engagement and other proactive and organizational citizenship behaviors (Orgambídez-Ramos et al., 2015).

Within the field of proactive behaviors, one area which merits further research is the concept of “job crafting” applied to the lives of priests. The concept of “job crafting” is an original approach to the changes that an employee may make to his or her job, without redesigning the entire position. A better understanding of this phenomenon can be gained by analyzing its structure in four factors, in accordance with the Spanish version of the Job Crafting Scale (Bakker et al., 2018). Job crafting involves: (1) increasing structural job resources: development of capacities, professional development, ongoing learning of aspects related to the job, the employee decides how best to do things, etc. (2) Decreasing hindering job demands: the employee strives to ensure that their job is mentally and emotionally less intense. (3) Increasing social job resources: by asking supervisors and colleagues for advice and feedback. And (4) increasing challenging job demands: employees become involved in new projects they find interesting, are not afraid of learning and trying out new things, make use of the times when the workload is lighter to think about new projects and take on challenging tasks, etc.

Job crafting is a model which links the physical, social and organizational demands made of an employee in their job (and which normally take a physical and psychological toll) with the resources available to them. Someone who crafts their job minimizes the impact of these demands and uses all the skills they have attained, thereby ensuring high job satisfaction and effective management of occupational stress. To enable this, in addition to a proactive personality, employees also need (as seen above) an organizational climate which offers opportunities for this type of behavior (Wingerden & Niks, 2017).

A priest who has the freedom, creativity, ability and initiative to design his own job will feel more satisfied, will identify more with his organization/Church and will be less vulnerable to work-related stress and burnout syndrome.

One last variable that is worth highlighting here is related to rest periods and self-care. As seen earlier, the absence of boundaries in priests’ lives, over-orientation toward the priestly role and the feeling of having too much demanded from them results in clergy finding it difficult to set time aside for themselves.

Research into a strategy known as “recovery” (Alcover, 2016; Demerouti et al., 2013) is of particular interest here. This strategy is linked to rest during holidays, weekends and days off, but in the case of Catholic clergy, it should be taken into account that being a priest is different from working in a secular profession and days off fall on different dates. Moreover, emphasis has recently been placed on the importance of daily recovery (during and after each working day). The aim of recovery is twofold: firstly, it seeks to minimize the effects of tension and stress, and secondly, from a more positive perspective, it aims to improve well-being.

Alcover (2016) calls attention to a fundamental aspect here: recovery occurs both outside the workspace (external recovery) and inside it, through either short pauses throughout the working day or regular changes of activity (internal recovery). The author distinguishes between semi-leisure activities (housework and personal care tasks), passive recreation (e.g., watching television) and active recreation (physical and social activities).

As the reviewed articles explain, while spiritual dryness and the absence of prayer are risk factors for experiencing occupational stress, spiritual well-being positively correlates with people’s physical and emotional health. Research indicates that religious experiences protect from stress and positively influence the immune system, cardiovascular health, mental health, and healthy habits, etc. (Chirico, 2016).

Furthermore, for priests, a strong sense of vocation implies experiencing pastoral activity as a source of well-being. Priests face numerous demands every day and their working days can be long. However, having a consistent vocation makes them feel motivated to carry out their work. The task gives meaning to their lives because it is a response to the transcendent dimension: “answering God’s call,” “identifying with the Good shepherd." Viewing and experiencing pastoral activity as a generous gift rather than an exhausting chore is an important protective factor against occupational stress (John Paul II, 1979).

In this sense, Christian prayer (like other meditation techniques, such as mindfulness) elicits a “relaxation response” incompatible with occupational stress. Chirico et al. (2020) carried out an experiment with 50 teachers from a catholic school. The experimental group received two short weekly prayer training sessions for two months, significantly reducing their burnout levels and improving their job satisfaction compared to the control group.

Other previous research insists on the benefits of meditative prayer and religious practice, which are especially effective in consecrated participants. Consecrated women report a greater frequency of personal prayer, church attendance and subjective religiosity, which correlates positively with greater job satisfaction, fewer absences due to illness, less psychopathology and greater commitment to work, etc. (Chirico et al., 2017c).

As stated in the “Method” section, due to the scarcity of references identified in this field and the variability of the methods used, this study has the important limitation of serving only as an exploratory approach to the subject under analysis. However, its modest nature is also its main virtue, in that it reveals all the possibilities which exist for future study and research.

Another limitation is that the reviewed research did not provide exactly the same data. Due to this, it would be interesting to conduct an original research, controlling the possible odd variables. Besides, data are collected from different locations, which may impact in the results. Despite these limitations, our scoping review tries to provide the most rigorous amount of data. This is one of the limitations of the scoping reviews; on the contrary, this may help us to explore the subject matter, and future research should benefit of this analysis for designing new approaches.

It seems clear that, in light of everything outlined above, more research is required into diocesan clergy and their experiences of work-related stress, bearing in mind a series of moderator variables such as relationship with their bishop and fellow priests, sociodemographic variables, identification with the organization and proactive behavior.

It would also be interesting to find evidence in support of the idea proposed by López (2012), who claimed that, among Catholic clergy, burnout and boreout syndromes tend to occur simultaneously as manifestations of work-related stress.

As we have seen, from a holistic perspective of health, finding times and spaces for prayer will lead to better overall health. Catholic dioceses, schools and hospitals, etc., can implement “schools of prayer and spirituality” for their consecrated and lay members. The efficacy of these programs could even be subsequently investigated with a view to improving them (Chirico & Magnavita, 2019).

Finally, in future research, it will be important to relate the impact of Covid-19 to occupational stress in priests. On the one hand, in such situations of crisis, people turn to spiritual comfort to help them deal with death and uncertainty about the future; yet on the other, priests (especially chaplains in hospitals, funeral homes and cemeteries, many of whom are elderly) face the burden of offering spiritual care, comfort in coping with grief and hope in the face of financial difficulties. Moreover, priests put their lives at risk by administering sacraments and spiritual care, both of which involve physical contact, and often do not feel respected by other health professionals or feel unprepared to offer their services online (Bramstedt, 2020). However, it should be remembered that sacraments (e.g., confession) cannot be administered online.
